# Gammaherpesvirus infection unveils exaggerated germinal center responses in an SR-BI-deficient host

**DOI:** 10.1128/jvi.00757-25

**Published:** 2025-05-30

**Authors:** Damon L. Schmalzriedt, Erika R. Johansen, Carlie A. Aurubin, Cade R. Rahlf, Bradey Stuart, Daisy Sahoo, Vera L. Tarakanova

**Affiliations:** 1 Department of Microbiology and Immunology, Medical College of Wisconsin 735651, Milwaukee, Wisconsin, USA; 2 Medical Scientist Training Program, Medical College of Wisconsin 5506, Milwaukee, Wisconsin, USA; 3 Department of Medicine, Medical College of Wisconsin 196253, Milwaukee, Wisconsin, USA; 4 Cardiovascular Center, Medical College of Wisconsin 601983, Milwaukee, Wisconsin, USA; 5 Cancer Center, Medical College of Wisconsin 166013, Milwaukee, Wisconsin, USA; The University of Arizona, Tucson, Arizona, USA

**Keywords:** germinal center response, SR-BI, cholesterol, gammaherpesvirus

## Abstract

Lipid metabolism has emerged as an important regulator of acute viral infections by affecting viral replication and the host immune response. In contrast, the role of host lipid metabolism during chronic viral infection has been less explored. The current study aims to define the role of scavenger receptor class B type I (SR-BI), a primary physiologic receptor for high-density lipoprotein (HDL), during natural gammaherpesvirus infection. Gammaherpesviruses are highly prevalent cancer-associated pathogens that induce and manipulate germinal center responses to establish life-long infection of B cells. Importantly, gammaherpesvirus-infected germinal center B cells are thought to be the target of viral transformation. In this study, we found that global SR-BI deficiency led to increased gammaherpesvirus lytic gene expression in the lungs during the acute stage of infection. Chronic gammaherpesvirus infection of the SR-BI-deficient host was associated with exaggerated germinal center responses and increased differentiation of self-reactive B cells that persisted during the long-term stage of chronic infection. Interestingly, SR-BI-deficient germinal center B cells, although more numerous, failed to support efficient gammaherpesvirus infection. The exaggerated germinal center response was also observed following immunization of the SR-BI-deficient host, unveiling the novel role of SR-BI as a negative regulator of physiological and gammaherpesvirus-driven germinal center responses.

**IMPORTANCE:**

Lipid metabolism affects diverse acute viral infections. In contrast, less is known about the effect of lipid metabolism on chronic virus infection, including in the context of an intact host. Host lipid homeostasis is maintained via a combination of endogenous lipid synthesis that takes place in most cell types and cellular interaction with exogenous, circulating lipids. This study focuses on defining the interaction between SR-BI, a primary HDL receptor, and natural gammaherpesvirus infection. We found that SR-BI deficiency led to increased expression of lytic gammaherpesvirus genes during acute gammaherpesvirus replication in the lungs. Importantly, chronic gammaherpesvirus infection unveiled the physiological role of SR-BI as a negative regulator of the germinal center response, a B cell differentiation process that is critical for physiological B cell responses and that is manipulated by gammaherpesviruses to establish chronic infection.

## INTRODUCTION

Gammaherpesviruses, such as human Epstein-Barr virus (EBV) and Kaposi’s sarcoma-associated herpesvirus (KSHV), establish lifelong infection and are associated with several malignancies and autoimmune disease (1, 2). Decreased control of chronic infection, as manifested by increased latent viral reservoir and viral reactivation from latency, precedes the development of malignancies (3–9). However, there is insufficient mechanistic understanding of the genetic and environmental factors that control chronic EBV or KSHV infection, in part due to the species specificity of human gammaherpesviruses. To overcome this limitation and evaluate host determinants of gammaherpesvirus control *in vivo*, this study utilizes murine gammaherpesvirus 68 (MHV68), a natural rodent pathogen that offers a powerful animal model of gammaherpesvirus infection. MHV68 shares significant genetic homology with EBV and KSHV (10–12) and recapitulates several core pathogenic strategies of human gammaherpesviruses, including infection of myeloid cells and B cells (13–19), induction of B cell differentiation to seed the viral latent reservoir (20, 21), and lymphoproliferative disease and oncogenesis in an immunocompromised host (9, 22).

Manipulation of B cell differentiation by gammaherpesviruses plays a key role in chronic gammaherpesvirus infection and pathogenesis (21, 23–27). Specifically, EBV and MHV68 infect naive B cells and drive differentiation of infected and bystander B cells into the germinal center response (21, 25, 27). Rapid proliferation of infected germinal center B cells leads to an increase in the viral latent reservoir, with further differentiation of infected germinal center B cells into memory B cells supporting lifelong latent infection. In contrast, differentiation of infected germinal center B cells into antibody-secreting plasma cells supports EBV and MHV68 reactivation (28, 29). Importantly, infection of germinal center B cells is thought to precede lymphomagenesis, as most EBV-positive lymphomas are of germinal center or post-germinal center origin (9). Despite the importance of the germinal center response in natural infection and pathogenesis, the mechanisms underlying manipulation of the germinal center response by EBV are poorly understood, largely due to the exquisite species specificity of human gammaherpesviruses and inability to model the germinal center response *in vitro*. Thus, the MHV68 experimental model has been instrumental in allowing identification of several host and viral factors that support or attenuate gammaherpesvirus-driven germinal center responses during chronic infection (23, 30–42).

Host lipid metabolism has emerged as an important regulator of lytic and latent gammaherpesvirus infection. Studies of the Lagunoff and Gewurz groups demonstrated the proviral role of endogenous fatty acid and cholesterol synthesis during EBV and KSHV infection of primary B cells and endothelial cells *in vitro* (43–45). The proviral role of endogenous lipid synthesis during gammaherpesvirus infection is conserved across species. as attenuation of endogenous cholesterol synthesis, whether iatrogenic (statin treatment) or physiological (increased expression of Liver X Receptors during infection), also decreases MHV68 lytic replication in primary macrophages (46, 47).

Host lipid homeostasis is maintained via a combination of endogenous, cell-intrinsic lipid metabolism and circulating host lipids, with many of those packaged with specialized proteins to form lipoproteins. The circulating lipoproteins interact with cognate receptors to mediate lipid exchange and activate signaling pathways downstream of respective receptors. Despite the rising prevalence of hypercholesterolemia and hyperlipidemia in the United States and their association with cardiovascular disease (48), the role of circulating lipoproteins and their receptors during natural gammaherpesvirus infection remains unknown.

In contrast to low-density lipoprotein-cholesterol (LDL-C), deemed as “bad” cholesterol due to its association with cardiovascular disease (49), HDL-cholesterol (HDL-C) is considered “good” cholesterol due to its overall cardioprotective role (50). However, significantly elevated HDL-C levels are also associated with cardiovascular disease via a poorly understood mechanism (51, 52). Under physiological conditions, HDL almost exclusively engages scavenger receptor class B type I (SR-BI), via interaction mediated by apolipoprotein A-I (apoA-I), the most abundant HDL protein (53). Importantly, several loss-of-function SR-BI mutations have been identified in humans with abnormally high HDL-C levels (reviewed in references 54, 55). However, the extent to which gammaherpesvirus infection is altered in these individuals has never been examined.

In this study, the role of SR-BI in acute and chronic gammaherpesvirus infection was defined using a mouse model of global SR-BI deficiency (*Scarb1*^-/-^, referred to as SR-BI^-/-^ in this manuscript) (56). Similar to that observed in humans with loss-of-function SR-BI mutations, SR-BI^-/-^ mice exhibit a selective increase in HDL-C levels (56). Although SR-BI^-/-^ mice fed a chow diet do not spontaneously develop cardiovascular disease, 20 weeks of a western diet leads to atherosclerosis in SR-BI^-/-^ mice (57). Furthermore, SR-BI deficiency synergizes with LDL-R deficiency to accelerate atherosclerosis (58). To eliminate the confounding effects of atherosclerosis on gammaherpesvirus infection, mice used in this study were maintained on the chow diet. Interestingly, we found distinct effects of global SR-BI expression during acute and chronic phases of gammaherpesvirus infection. Although the expression of lytic MHV68 genes was elevated in the lungs of acutely infected SR-BI^-/-^ mice, latent infection of germinal center B cells was less efficient. Furthermore, global SR-BI deficiency led to increased MHV68-driven germinal center response. The exaggerated germinal center response observed in MHV68-infected SR-BI^-/-^ mice was not limited to gammaherpesvirus infection, as immunization with sheep red blood cells also elicited increased germinal center response in SR-BI^-/-^ mice. ApoA-I deficiency, characterized by significantly reduced HDL-C levels (59), was associated with modest attenuation of gammaherpesvirus-driven germinal center response, suggesting that ligation of SR-BI by HDL is not necessary for SR-BI-dependent attenuation of germinal center responses. Importantly, exaggerated germinal center responses and differentiation of self-reactive B cells observed in SR-BI^-/-^ mice during the establishment of MHV68 latency were not transient and instead persisted in the long-term stage of infection.

## RESULTS

### Global SR-BI deficiency leads to elevated MHV68 lytic gene expression but does not affect IFN responses during acute infection

**Fig 1:**
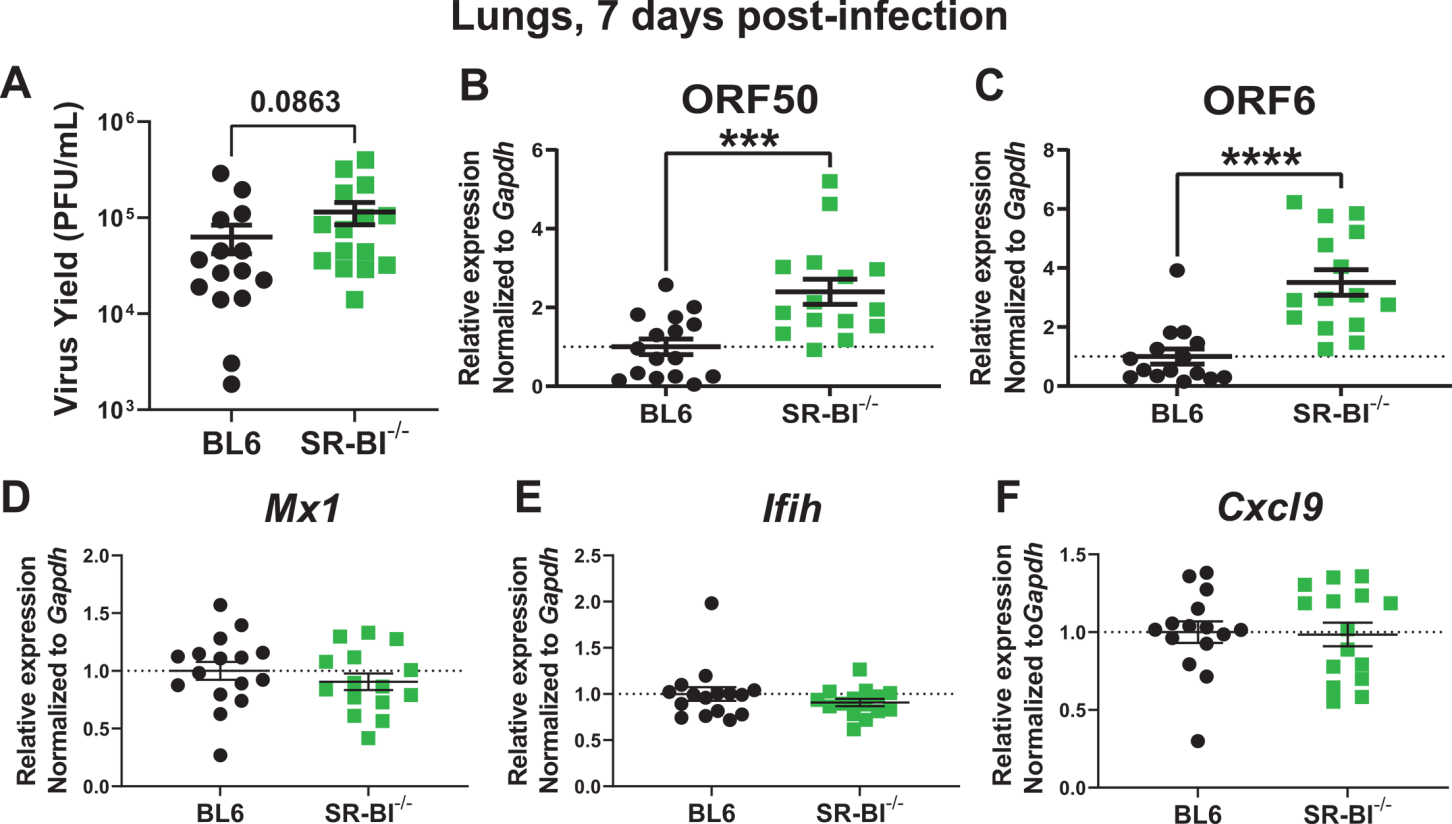
Global SR-BI deficiency leads to elevated MHV68 lytic gene expression but does not affect IFN responses during acute infection. BL6 and SRB1^-/-^ mice were intranasally infected with 10,000 PFU of MHV68. Lungs were harvested at 7 days post-infection. (**A**) Lung homogenates were subjected to plaque assay to determine MHV68 lytic titers. (B–F) RNA was isolated from individual lungs at 7 days post-infection and subjected to qRT-PCR analysis for relative mRNA expression of (**B**) MHV68 *orf50,* (**C**) MHV68 *orf6,* (**D**) *Mx1,* (**E**) *Ifih,* and (**F**) *Cxcl9*. Relative gene expression was normalized to corresponding *Gapdh* expression levels and further normalized to the average of the BL6 group (set at one and marked by the dotted line). Each symbol represents an individual mouse. Here and throughout all figures, error bars represent the standard error of the mean (SEM). ***P < 0.001, ****P < 0.0001.

Following intranasal inoculation of a naïve host, MHV68 undergoes a brief period of acute replication, with lung titers peaking at 4–7 days, and infectious virions largely cleared around 10–12 days post-infection (60). When peak MHV68 lytic replication was assessed in the lungs, SR-BI^-/-^ mice displayed slightly elevated MHV68 titers compared with BL6 mice, with the difference not reaching statistical significance (Fig. 1A). However, mRNA levels of lytic MHV68 genes (*orf50* and *orf6*, encoding lytic switch transcription factor and single stranded DNA binding protein, respectively) were significantly increased in SR-BI^-/-^ lungs (Fig. 1B and C). Thus, global SR-BI deficiency resulted in increased lytic MHV68 gene expression during acute infection.

Type I interferon (IFN) expression and signaling play an important role in the control of acute MHV68 replication and infected host survival (61, 62). Furthermore, STAT1 activation downstream of type I and II IFN receptors directly attenuates MHV68 orf50 expression (63, 64). Interestingly, SR-BI ligation by ApoA-I mimetic, although not sufficient to induce type I IFN expression, boosted type I IFN signaling and antiviral activity in L929 fibroblasts infected with encephalomyocarditis virus (EMCV) (65). Having observed increased expression of MHV68 lytic genes in the SR-BI^-/-^ lungs, type I IFN signaling was assessed next by measuring expression of two IFN-stimulated genes (ISGs), *Mx1* and *Ifih* (66). In contrast to that observed in EMCV-infected L929 cell line (65), there was no difference in mRNA levels of Mx1 or Ifih in lungs of MHV68-infected BL6 and SR-BI^-/-^ mice (Fig. 1D and E). Similarly, mRNA levels of Cxcl9, an ISG preferentially induced by type II IFN, were similar in infected BL6 and SR-BI^-/-^ lungs (Fig. 1F). In summary, although type I and II IFN signaling in the lung was not affected by global SR-BI deficiency, MHV68 lytic gene expression was elevated in acutely infected SR-BI^-/-^ lungs.

### Global SR-BI deficiency leads to attenuated MHV68 reactivation in the peritoneal cavity without affecting peritoneal and splenic latent reservoirs and viral reactivation from the splenocytes

**Fig 2:**
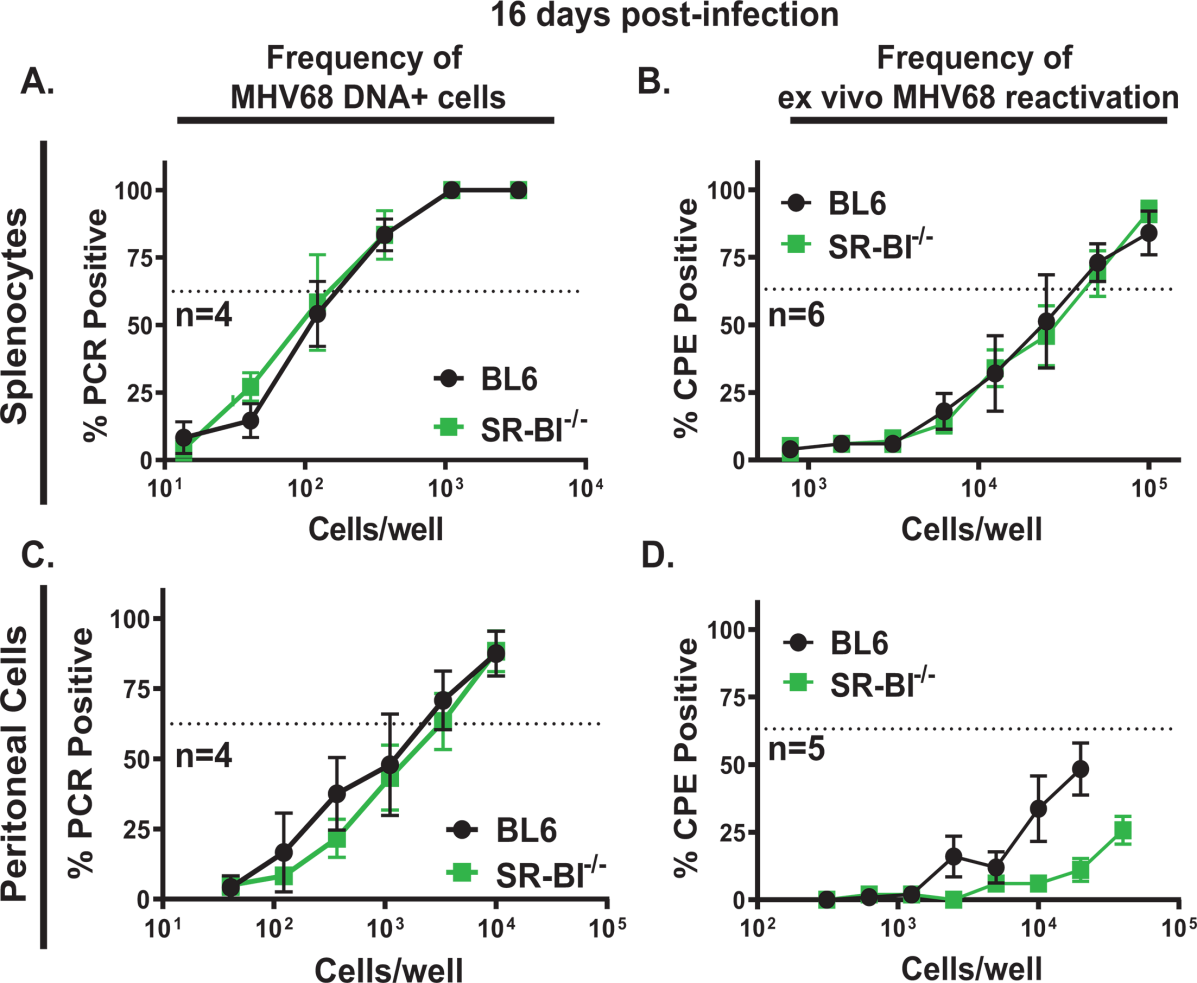
Global SR-BI deficiency leads to attenuated MHV68 reactivation in the peritoneal cavity without affecting peritoneal and splenic latent reservoirs and viral reactivation from the splenocytes. BL6 and SR-BI^-/-^ mice were infected as in Fig. 1 and analyzed at 16 days post-infection. Splenocytes (A, B) and peritoneal cells (C, D) were pooled from 3 to 5 animals/group in each study, and cell suspensions were subjected to limiting dilution assays to define the frequency of MHV68 DNA + cells (A, C) and frequency of *ex vivo* reactivation (B, D). Data were pooled from the indicated number (N) of independent studies. In the limiting dilution assays, the dotted line is drawn at 63.2%, and the x-coordinate of the intersection of this line with the sigmoid graph represents the inverse of the frequency of positive events.

Concurrent with the infectious MHV68 clearance from the lungs, there is a rise in the latent viral reservoir in the spleen, with the peak latent reservoir observed at 16 days post-infection. In contrast to lung epithelial cells and macrophages that primarily support acute MHV68 replication (67), B cells, particularly germinal center B cells, host the majority of the latent MHV68 reservoir in the spleen at 16 days post-infection (24). Differentiation of infected germinal center B cells into plasma cells is primarily responsible for MHV68 reactivation from splenocytes (29). Interestingly, despite elevated levels of lytic MHV68 gene expression observed in acutely infected SR-BI^-/-^ lungs, the frequencies of latently infected splenocytes and *ex vivo* MHV68 reactivation were similar in SR-BI^-/-^ and BL6 spleens at 16 days post-infection (Fig. 2A and B).

Splenic MHV68 latency is primarily supported by germinal center B cells, which represent the B-2 B cell lineage. In contrast, following intranasal inoculation, MHV68 preferentially infects peritoneal B cells that are of the B-1 B cell lineage (15). Interestingly, although global SR-BI deficiency had no effect on the latent MHV68 reservoir in the peritoneal cavity, the frequency of *ex vivo* reactivation from peritoneal cells was decreased in SR-BI^-/-^ mice (Fig. 2C and D). In summary, global SR-BI deficiency led to the attenuation of MHV68 reactivation from peritoneal cells but had no effect on viral reactivation in the spleen or the magnitude of overall latent reservoirs in the spleen or peritoneal cavity.

### Global SR-BI deficiency leads to exaggerated MHV68-driven germinal center response

MHV68, like EBV, manipulates the germinal center response to establish its latent reservoir. Specifically, both infected and bystander naïve B cells are driven into the germinal center, where B cells undergo robust proliferation that leads to amplification of the MHV68 latent reservoir (26). Similar to that observed for physiological germinal center responses, MHV68-driven germinal center response is critically supported by the CD4^+^ T follicular helper cells (23, 68). Although germinal center responses have not been examined in SR-BI^-/-^ mice, splenic B cells from unmanipulated aged (>20 weeks) SR-BI^-/-^ mice show increased activation and proliferation, measured by CD69 expression and BrdU incorporation, respectively (69). Further aging of SR-BI^-/-^ mice (>30 weeks of age) leads to the generation of self-reactive antibodies and deposition of immune complexes in the glomeruli, even when the mice are maintained on a chow diet in the absence of any experimental manipulations (69). The mechanisms underlying increased baseline B cell activation and autoimmune disease in aged SR-BI^-/-^ mice remain undefined.

Given the differences in the age of SR-BI^-/-^ mice in the published (>20 weeks [69]) and this study (6 weeks at infection), immunophenotyping of splenic B cells was performed in unmanipulated SR-BI^-/-^ mice of the same age as those analyzed at 16 days post-MHV68 infection studies (8-9 weeks of age) using gating strategies outlined in Fig. S1. Despite the increased number of splenocytes observed in young SR-BI^-/-^ mice, the proportion of splenocytes represented by CD19^+^B220^+^ B cells was decreased, without change in the total B cell numbers (Fig. S2A and B), indicating that the observed increase in splenocyte numbers was driven by a non-B cell immune population. Interestingly, the proportions of follicular and marginal zone B cells were decreased in the overall splenic B cell population of SR-BI^-/-^ mice, including a decrease in absolute marginal zone B cell numbers (Fig. S2C and D). In contrast, transitional B cell populations, including T1 and T2 that give rise to follicular and marginal zone B cells, were either unchanged or increased in SR-BI^-/-^ spleens (Fig. S2E through G).

**Fig 3:**
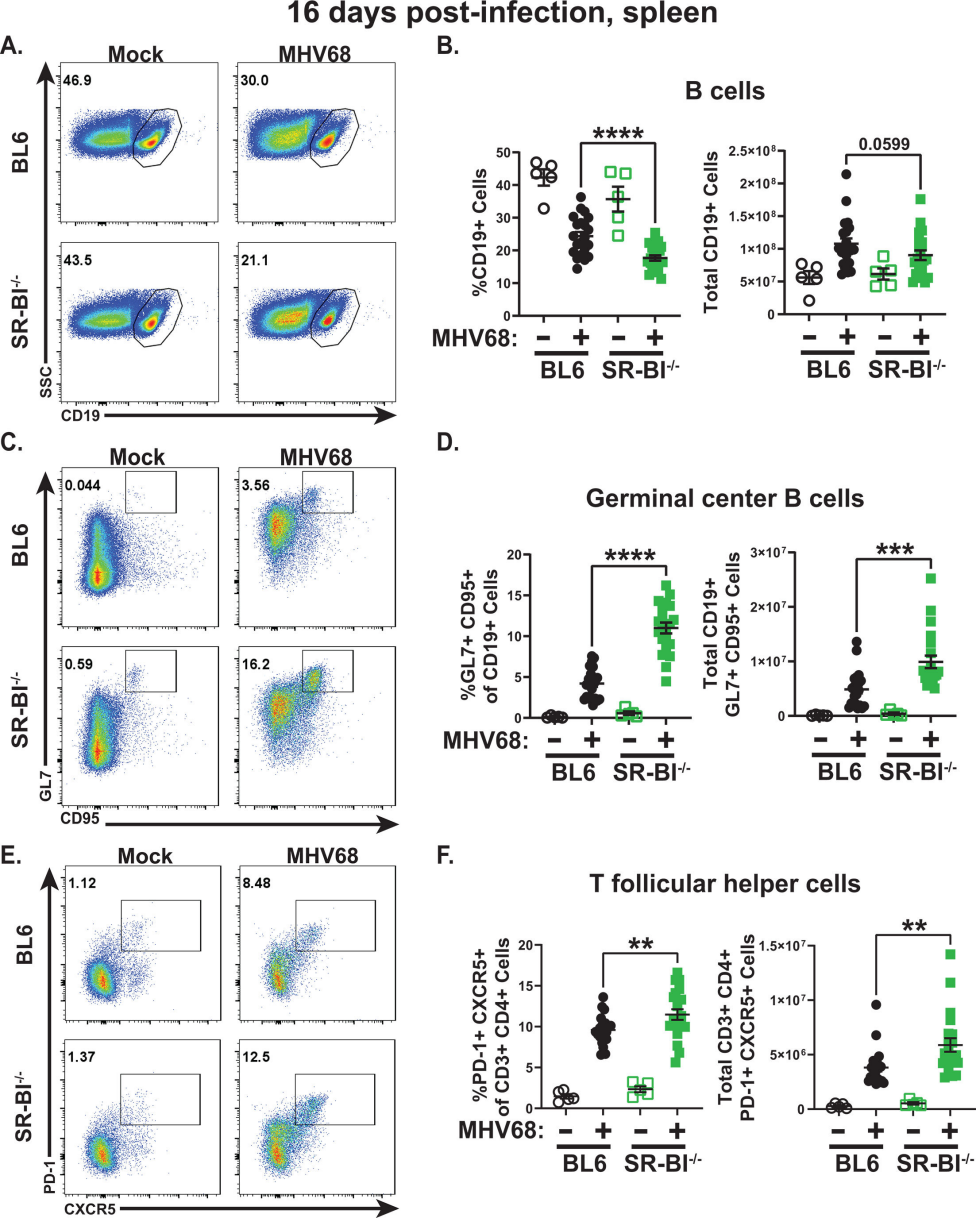
Global SR-BI deficiency leads to exaggerated MHV68-driven germinal center response. BL6 and SR-BI^-/-^ mice were infected as in Fig. 1 and analyzed at 16 days post-infection. (A, B). Splenic B cells, defined as CD19^+^ (representative flow plots in panel **A**), were measured by flow cytometry, with proportion and absolute cell number per spleen shown. (C, D). Splenic germinal center B cells defined as CD19^+^GL7^+^CD95^+^ were measured (representative flow plot in panel **C**) with proportion and absolute cell number per spleen shown. (E, F). Splenic T follicular helper cells defined as CD3^+^CD4^+^PD-1^+^CXCR5^+^ were measured (representative flow plot in E) with proportion and absolute cell number shown. Each symbol represents an individual animal. **P < 0.01, ***P < 0.001, ****P < 0.0001.

Having defined the effect of global SR-BI expression on the splenic B cell populations in naïve young mice, MHV68-driven B cell differentiation was assessed next. The decrease in the proportion of splenocytes represented by B cells that was observed in naïve SR-BI^-/-^ mice remained pronounced following MHV68 infection, with the absolute number of splenic SR-BI^-/-^ B cells trending toward a decrease, but not reaching statistical significance in infected mice (Fig. 3B, gating strategy in Fig. 3A). Consistent with increased baseline B cell activation previously reported in aged SR-BI^-/-^ mice, the proportion, but not absolute number of germinal center B cells, was elevated in the spleens of mock-infected young SR-BI^-/-^ mice compared with BL6 counterparts (*P* = 0.03, Fig. 3D, gating strategy in Fig. 3C). MHV68 infection further exacerbated the differences in germinal center B cells observed at baseline, with both proportion and the absolute number of germinal center B cells significantly increased in infected SR-BI^-/-^ mice (Fig. 3D). Similar to that observed for germinal center B cells, the proportion, but not number of T follicular helper cells, was increased at baseline in SR-BI^-/-^ spleens (*P* = 0.04, Fig. 3F, gating strategy in Fig. 3E), and this difference was further exacerbated at 16 days post-MHV68 infection (Fig. 3F). In summary, SR-BI deficiency led to increased baseline germinal center responses, a phenotype that was exacerbated at 16 days post-MHV68 infection.

### Global SR-BI deficiency leads to increased germinal center response following sheep red blood cell immunization

**Fig 4:**
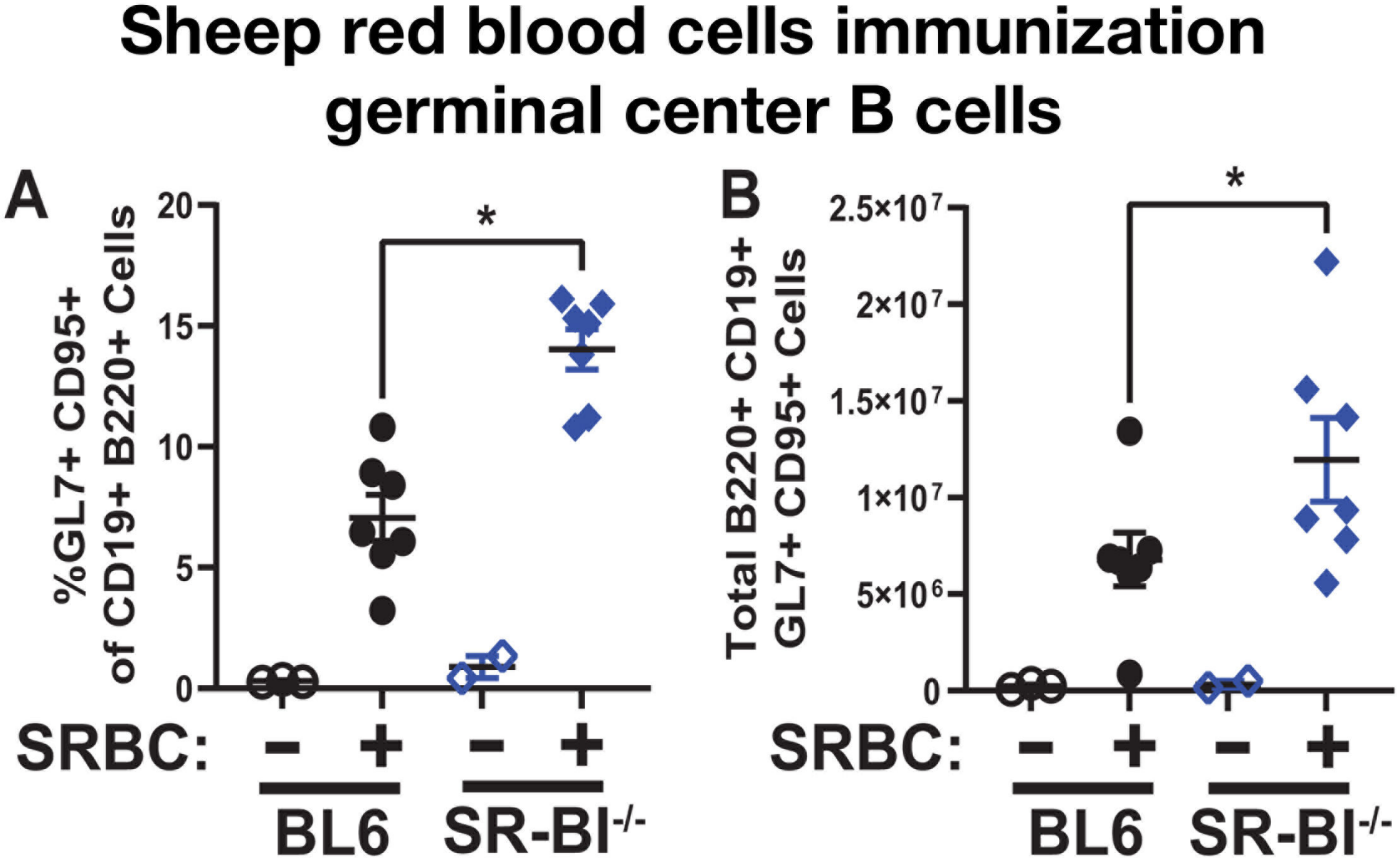
Global SR-BI deficiency leads to increased germinal center response following sheep red blood cells immunization. BL6 and SR-BI^-/-^ mice were intraperitoneally injected with sheep red blood cells and analyzed 7–9 days post-immunization. Splenic germinal center B cells defined as CD19^+^GL7^+^CD95^+^ were measured (representative flow plot as in Fig. 3C) with proportion (**A**) and absolute cell number (**B**) shown. Each symbol represents an individual animal. *P < 0.05.

Gammaherpesviruses both induce and manipulate germinal center responses to establish latent infection. Correspondingly, the host has evolved mechanisms that selectively counteract gammaherpesvirus-driven, but not physiological B cell differentiation, as we previously demonstrated for Interferon Regulatory Factor 1 (70). Having observed exaggerated germinal center response driven by MHV68 infection in SR-BI^-/-^ mice, the specificity of the observed germinal center phenotype for gammaherpesvirus infection was assessed next using immunization with sheep red blood cells (SRBC). Interestingly, the proportion and absolute numbers of germinal center B cells were also elevated in SR-BI^-/-^ mice following SRBC immunization (Fig. 4A and B), indicating that the exaggerated germinal center response observed in MHV68-infected SR-BI^-/-^ mice was not unique to gammaherpesvirus infection.

### Latent MHV68 infection of splenic germinal center B cells is attenuated in SR-BI^-/-^ mice

**Fig 5:**
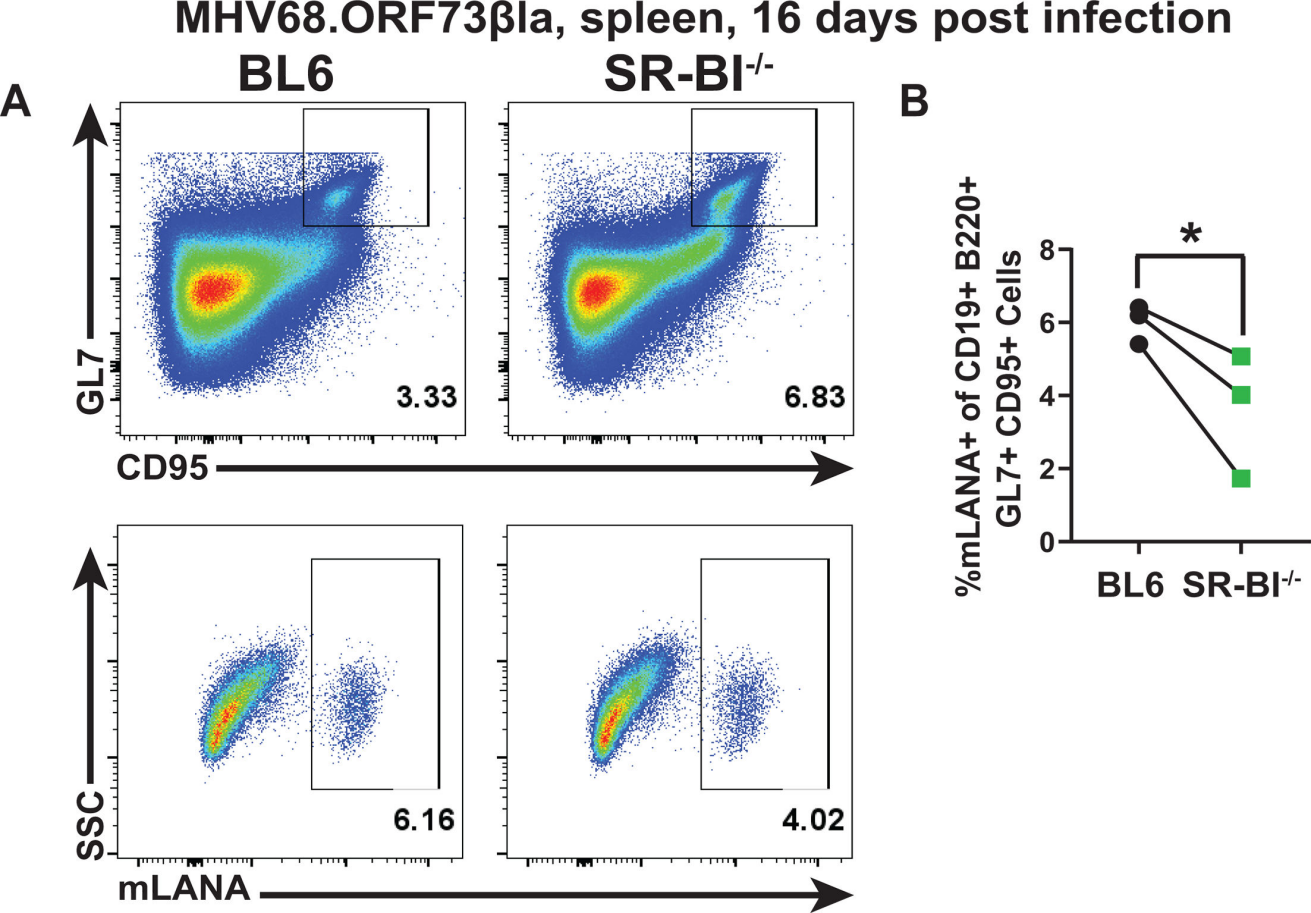
MHV68 infection of splenic germinal center B cells is attenuated in SR-BI^-/-^ mice. BL6 and SR-BI^-/-^ mice were intranasally infected with 10,000 PFU of MHV68.ORF73βla reporter virus, spleens harvested at 16 days post-infection, and pooled from three animals within each group within each independent experiment. Infected cells were defined as positive for cleaved CCF2 β-lactamase substrate (indicated as CCF2^+^). (**A**) Representative gating strategy for GL7^+^CD95^+^ germinal center B cells pregated on CD19^+^B220^+^ splenocytes and postgated on population positive for cleaved CCF2 substrate (CCF2^+^). (**B**) Percent of infected germinal center B cells. In panel B, each symbol represents analysis of splenocytes pooled from 3 to 5 mice/genotype in a single experiment, and the connecting lines represent paired observations within a single study. Results of 3 studies are shown. *P < 0.05.

Given that the germinal center B cells support the majority of the latent splenic MHV68 reservoir at 16 days post-infection, an increased MHV68-driven germinal center response (Fig. 3C and D) without a corresponding increase in the splenic latent reservoir of SR-BI^-/-^ mice (Fig. 2A) suggested that the frequency of latently infected germinal center B cells was decreased in SR-BI^-/-^ mice. To measure the frequency of infected germinal center B cells, BL6 and SR-BI^-/-^ mice were infected with MHV68.ORF73βLa, a reporter virus encoding beta-lactamase fused to MHV68 mLANA, with beta-lactamase enzymatic activity marking infected cells (30, 71). Effective loading of CCF2, as measured by the emission of uncleaved substrate in germinal center B cells, was monitored as in Fig. S3. As predicted, the frequency of beta-lactamase positive, infected germinal center B cells was significantly decreased in SR-BI^-/-^ mice (Fig. 5B, gating strategy in Fig. 5A), indicating that germinal center B cells of SR-BI^-/-^ mice, although increased in abundance, were less capable of supporting MHV68 latent infection.

### Global SR-BI deficiency leads to a selective increase in MHV68-driven self-reactive, but not virus-specific antibodies, at 16 days post-infection

In addition to the physiological B cell differentiation that generates virus-specific antibodies, EBV and MHV68 drive a germinal center response that uniquely generates high titers of antibodies reactive against self- and foreign-species antigens (72, 73). Correspondingly, EBV and MHV68 preferentially establish long-term infection in B cells that do not express a gammaherpesvirus-specific antibody (74, 75).

The kinetics of physiological versus self-reactive B cell differentiation are distinct during gammaherpesvirus infection. In contrast to the robust increase in self-reactive antibodies that peak at 14–16 days post-MHV68 infection (73), antiviral antibody titers rise with slower kinetics, with the peak of anti-MHV68 IgG titers not observed until 30 days post-infection. Similarly, a diagnostic test for recent EBV infection in humans relies on the detection of antibodies against horse red blood cells, with the class-switched anti-EBV titers arising weeks later (76).

**Fig 6:**
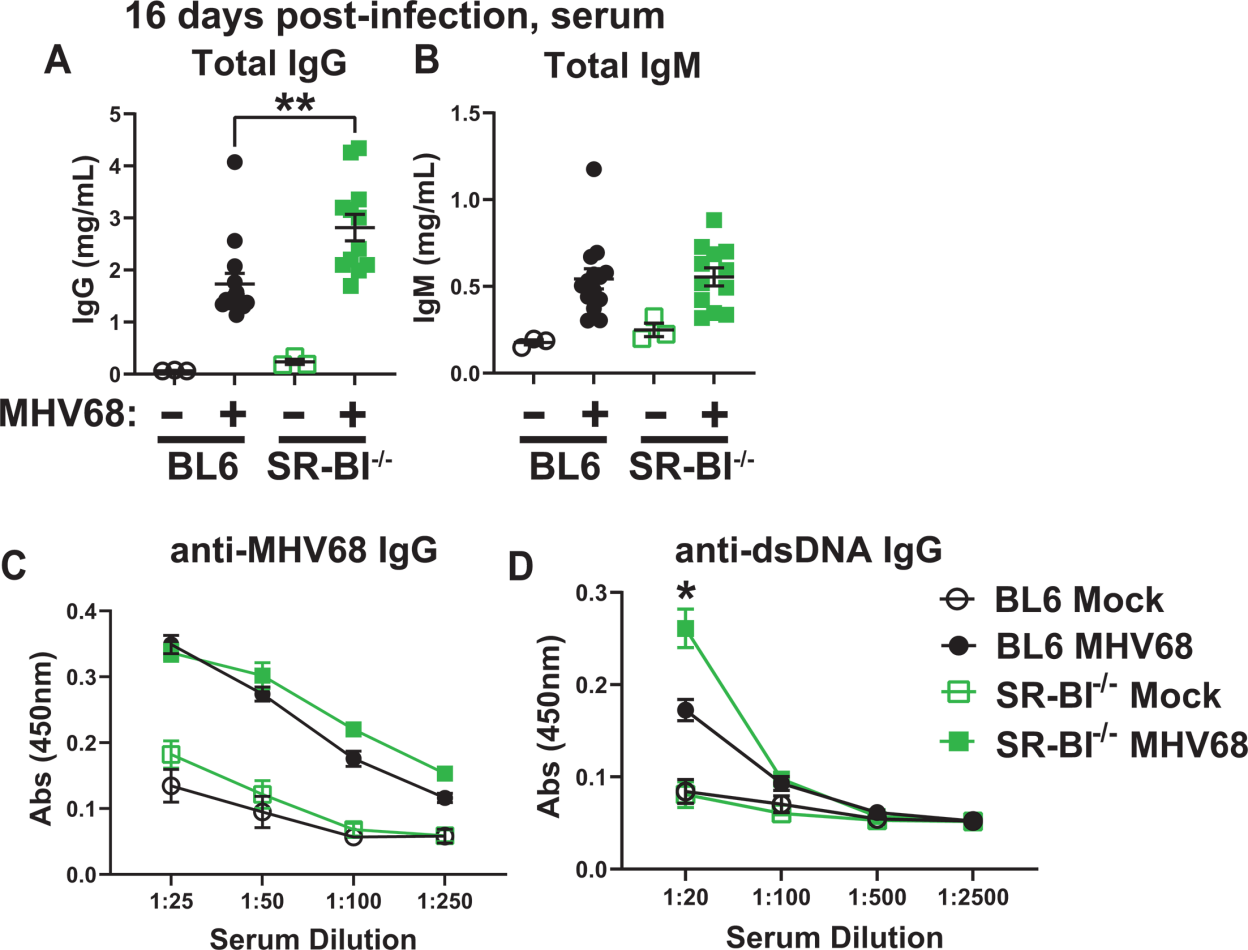
Global SR-BI deficiency leads to a selective increase in MHV68-driven self-reactive, but not virus-specific antibodies at 16 days post-infection. BL6 and SR-BI^-/-^ mice were mock- or MHV68-infected as in Fig. 1, sera were collected at 16 days post-infection, and subjected to ELISA to measure levels of total IgG (**A**), total IgM (**B**), anti-MHV68 IgG (**C**), and anti-double stranded DNA IgG (**D**). Each symbol in panels A and B represents an individual animal. In panel C, sera were pooled from three mock-infected mice and 12-14 MHV68-infected mice for each group. In panel D, sera were pooled from 4 to 5 mock-infected mice and 13-15 MHV68-infected mice per group, with averages and standard error of measurement shown. *P < 0.05; **P < 0.01.

Having observed increased germinal center responses in MHV68-infected SR-BI^-/-^ mice, antibody titers were measured next. Consistent with elevated numbers of germinal center B cells, MHV68-infected SR-BI^-/-^ mice demonstrated increased serum titers of total IgG, but not IgM, at 16 days post-infection (Fig. 6A and B). However, the elevated levels of total IgG were not driven by increased levels of anti-MHV68 IgG, which remained the same in BL6 and SR-BI^-/-^ mice (Fig. 6C).

MHV68 infection drives an increase in self-reactive antibodies with diverse specificities; however, antibodies against double-stranded DNA (dsDNA) are induced in a majority of infected mice at 16 days post-infection and represent a reliable assessment of MHV68-driven differentiation of self-reactive B cells (73, 77). Consistent with a previous study demonstrating that self-reactive antibody titers do not increase until 30 + weeks of age in SR-BI^-/-^ mice, mock-infected young SR-BI^-/-^ mice did not display elevated titers of anti-dsDNA IgG compared with age-matched controls (Fig. 6D) (69). In contrast, MHV68-driven titers of anti-dsDNA IgG were significantly higher in SR-BI^-/-^ mice (Fig. 6D), suggesting that MHV68 infection accelerates the development of autoimmune antibodies in SR-BI^-/-^ mice.

### Global SR-BI deficiency does not affect the levels of regulatory CD4 T cells but instead leads to increased numbers of MHV68-specific CD8 T cells

**Fig 7:**
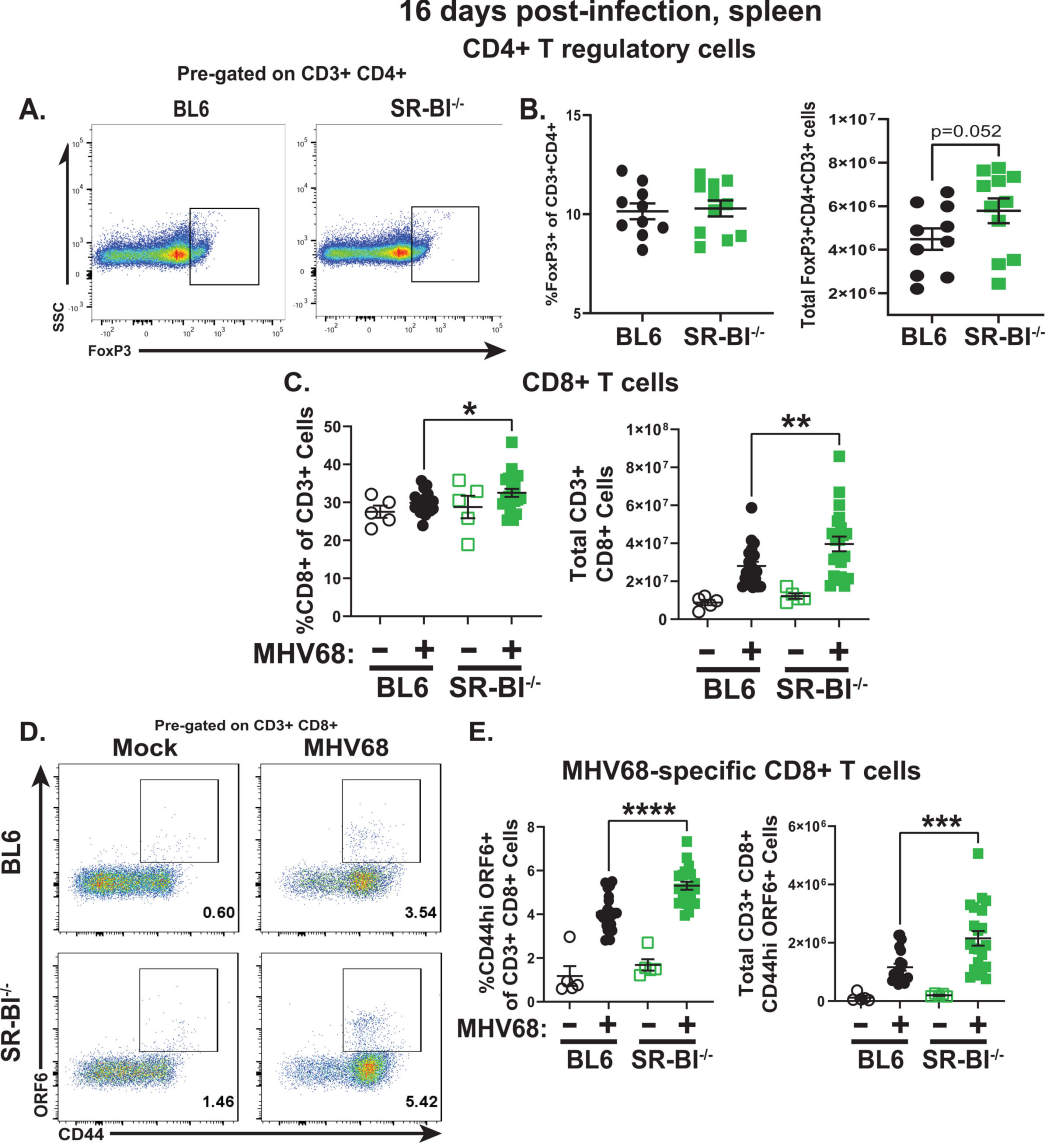
Global SR-BI deficiency does not affect levels of regulatory CD4 T cells but instead leads to increased numbers of MHV68-specific CD8 T cells. BL6 and SR-BI^-/-^ mice were infected as in Fig. 1 and analyzed at 16 days post-infection. (**A, B**) Splenic T regulatory cells, defined as CD3^+^CD4^+^FoxP3^+^ (representative flow plots in A), were measured by flow cytometry with proportion and absolute cell number shown in panel **B**. (**C**) Proportion and absolute numbers of splenic CD3^+^CD8^+^ T cells. (**D, E**) Splenic MHV68-specific CD8 T cells defined as CD3^+^CD8^+^CD44^hi^, also staining positive with MHV68 orf6 MHC-I tetramer, were measured (representative flow plot in D) with proportion and absolute cell number shown in panel **E**. Each symbol represents an individual animal. *P < 0.05, **P < 0.01, ***P < 0.001, ****P < 0.0001.

SR-BI facilitates HDL-cholesterol influx in endocrine cells, increasing the intracellular pool of cholesterol that serves as a substrate for corticosteroid synthesis (78, 79). Although baseline corticosteroid levels are similar in BL6 and SR-BI^-/-^ mice, increased corticosteroid production in response to *Klebsiella pneumoniae* infection is blunted in SR-BI^-/-^ mice (80). Because the immunosuppressive function of corticosteroids depends on the presence of regulatory CD4+ T cells (81), and given the increased immune activation observed in MHV68-infected SR-BI^-/-^ mice, regulatory CD4+ T cells were measured next. The proportion and number of CD4+ T cells represented by FoxP3+ regulatory T cells were similar in MHV68-infected BL6 and SR-BI^-/-^ mice, with the number of splenic CD4+ regulatory T cells trending towards increased in the latter group (Fig. 7A and B). Thus, SR-BI deficiency did not decrease the abundance of regulatory CD4+ T cells during chronic MHV68 infection.

Although CD8 T cell deficiency has a minimal, if any, effect on the establishment of splenic MHV68 latent reservoir at 16 days post-infection (82), increased numbers of MHV68-specific CD8+ T cells observed in *IL-10*^-/-^ mice are associated with attenuated MHV68 latent reservoir (83). Furthermore, CD8 T cells are important for the control of chronic MHV68 infection in the peritoneal cavity (82). Having observed decreased frequency of MHV68+ germinal center B cells in SR-BI^-/-^ mice and decreased MHV68 reactivation from peritoneal cells, CD8 T cell responses were measured next. Although the baseline levels of CD8 T cells were not altered by SR-BI deficiency, both the proportion and absolute numbers of splenic CD8 T cells were increased in MHV68-infected SR-BI^-/-^ mice (Fig. 7C). This increase in total CD8 T cells was mirrored by an increase in the proportion and absolute number of CD8 T cells specific for the immunodominant MHC-I epitope encoded by MHV68 orf6 (Fig. 7D and E). Thus, global SR-BI deficiency led to an increase in total and MHV68-specific CD8 T cell numbers following MHV68 infection.

### ApoA-I global deficiency leads to attenuated germinal center response during chronic MHV68 infection

**Fig 8:**
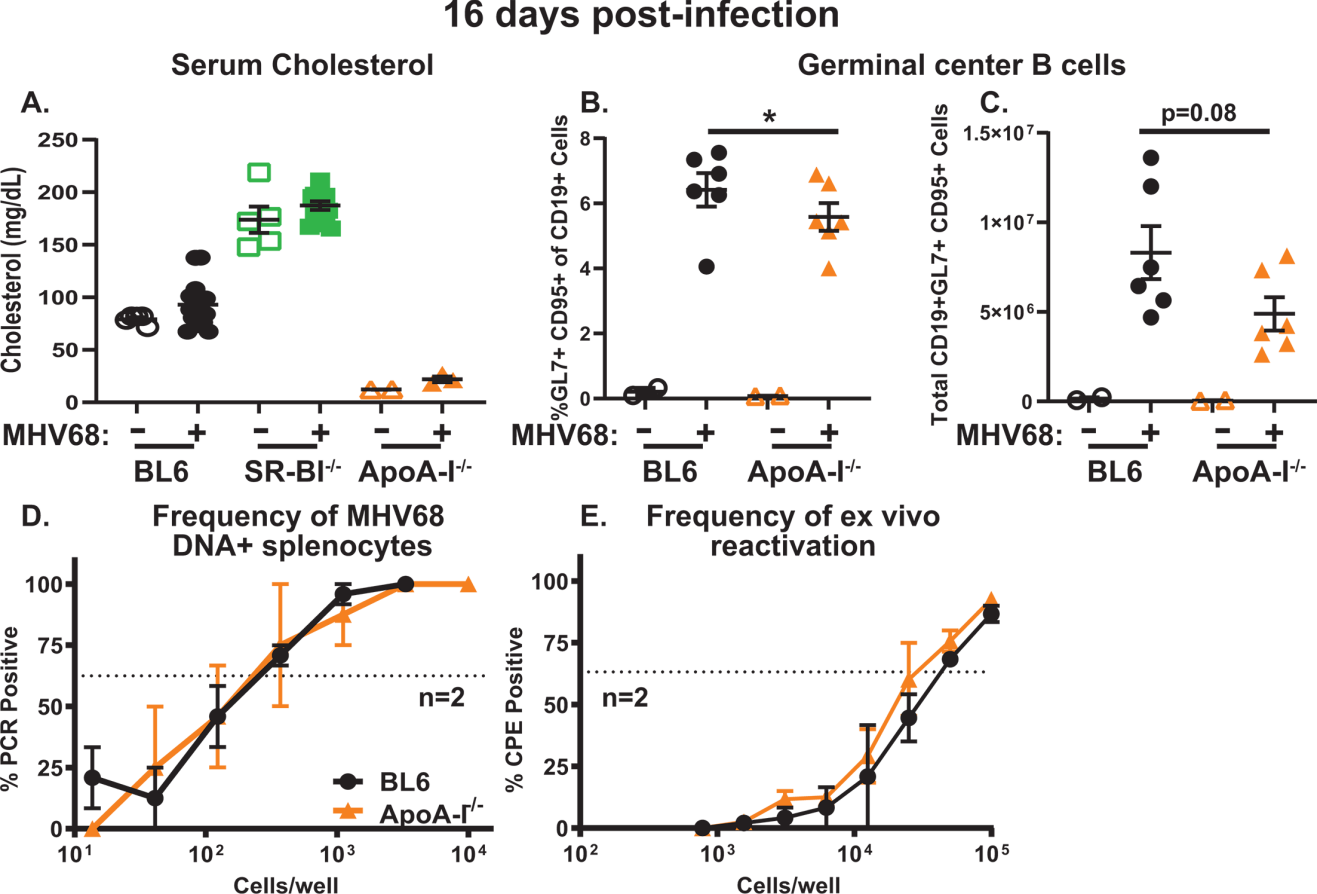
ApoA-I global deficiency leads to attenuated germinal center response during chronic MHV68 infection. BL6, SR-BI^-/-^, and ApoA-I^-/-^ mice were infected as in Fig. 1 and analyzed at 16 days post-infection. (**A**) Total serum cholesterol levels. (B, C) Germinal center B cells were identified and quantified as in Fig. 3 with proportion (**B**) and absolute numbers (**C**) shown. Each symbol represents an individual animal. (D, E) Frequency of MHV68 DNA + splenocytes and *ex vivo* reactivation was measured as in Fig. 2. The results were pooled from two independent studies, with splenocytes from three animals/group pooled in each study prior to limiting dilution analyses. *P < 0.05.

Serum cholesterol in most humans is primarily packaged as LDL-C, with a smaller proportion of the overall serum cholesterol levels associated with the HDL lipoproteins. In contrast, serum cholesterol of laboratory mice is primarily packaged as HDL-C (84). Despite these baseline differences, human and mouse SR-BI insufficiency results in a significant increase in total serum cholesterol that is primarily driven by increases in HDL-C levels, with the increase maintained during chronic MHV68 infection (Fig. 8A) (56). Therefore, the exaggerated B cell differentiation observed in SR-BI^-/-^ mice could be due to the lack of SR-BI ligation with HDL (due to the absence of SR-BI expression) and/or engagement of other lipoprotein receptors by increased HDL levels.

To gain insight into the mechanism underlying an exaggerated germinal center response observed in MHV68-infected SR-BI^-/-^ mice, a mouse model of apolipoprotein AI (ApoA-I) deficiency was tested next. Under normal physiological conditions, HDL almost exclusively engages SR-BI via interaction mediated by ApoA-I, the most abundant HDL apolipoprotein (53). Correspondingly, ApoA-I deficiency in mice is associated with decreased total serum cholesterol due to nearly absent HDL-C, with the decreased serum cholesterol levels maintained during chronic MHV68 infection (Fig. 8A) (59). Interestingly, the proportion of germinal center B cells was decreased in MHV68-infected *Apoa-I*^-/-^ compared with control mice, with a trending but not statistically significant decrease in the absolute number of germinal center B cells (Fig. 8B and C). Similar to that observed for SR-BI^-/-^ mice, splenic latent reservoir and *ex vivo* reactivation were comparable in control and *Apoa-I*^-/-^ mice at 16 days post-infection (Fig. 8D and E). In summary, global ApoA-I deficiency was associated with modest attenuation of MHV68-driven germinal center B cell abundance without affecting parameters of chronic infection in the spleen.

### Exaggerated MHV68-driven B cell differentiation persists in long-term infected SR-BI^-/-^ mice

**Fig 9:**
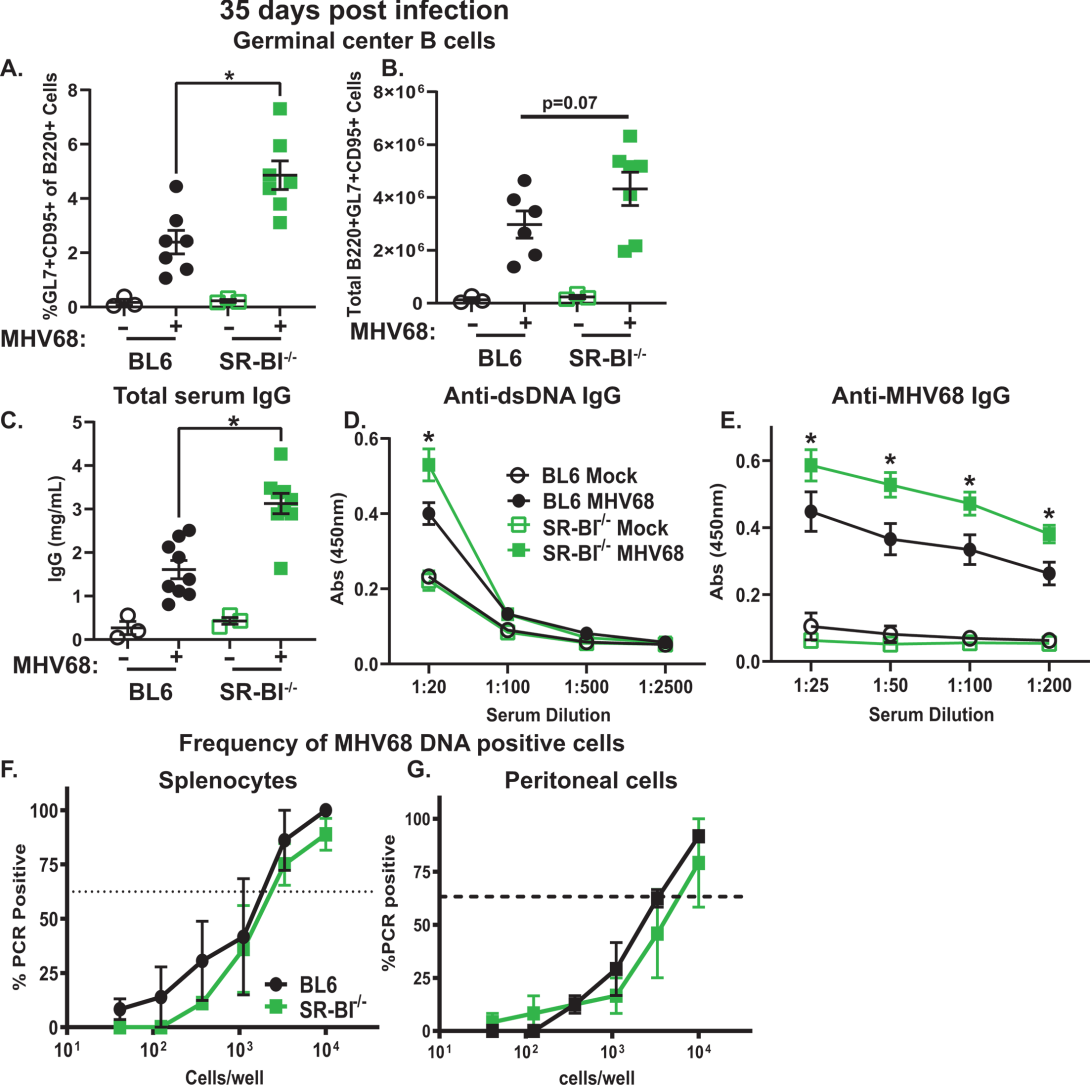
Exaggerated MHV68-driven B cell differentiation persists in long-term infected SR-BI*^-/-^* mice. BL6 and SR-BI^-/-^ mice were infected as in Fig. 1 and analyzed at 35 days post-infection. (A, B) Germinal center B cells were measured as in Fig. 3. (**C**) Total serum IgG was measured as in Fig. 6. (A through C) Each symbol represents an individual animal. (D, E) Serum IgG titers against dsDNA and MHV68 were measured as in Fig. 6, data were pooled from three mock-infected mice/group and nine MHV68-infected mice/group for each antibody titer. (F, G) Frequency of MHV68 DNA + splenocytes (**F**) and peritoneal cells (**G**) was determined as in Fig. 2. Data were pooled from 2 to 3 independent studies.

In contrast to that observed during the establishment of chronic infection at 16 days, the MHV68 latent reservoir in the spleen contracts and stabilizes by 35 days post-infection, with little, if any, viral reactivation observed. Compared with the peak MHV68-driven germinal center response observed at 16 days post-infection, the numbers of germinal center B cells also decrease in long-term infected mice. Accordingly, the proportion of germinal center B cells decreased from ~5% to ~2% in BL6 mice between 16 and 35 days post-MHV68 infection (compare Fig. 3D and 9A). However, the proportion of germinal center B cells remained elevated in long-term infected SR-BI^-/-^ mice, with a trending increase in the absolute number of germinal center B cells that did not reach statistical significance (Fig. 9A and B). This sustained increase in MHV68-driven germinal center response continued to support elevated levels of total and double-stranded DNA (dsDNA)-specific serum IgG, similar to that observed during the establishment of chronic infection (Fig. 9C and D). Interestingly, and in contrast to that observed at 16 days post-infection, long-term infected SR-BI^-/-^ mice displayed higher titers of MHV68-specific IgG compared with BL6 mice (Fig. 9E). Despite the increase in titers of MHV68-specific antibodies, the latent MHV68 reservoir was similar in the spleens and peritoneal cavity of long-term infected BL6 and SR-BI^-/-^ mice (Fig. 9F and G), with similarly low levels of persistent MHV68 replication observed in the lungs (data not shown). In summary, long-term MHV68 infection sustained exaggerated differentiation of B cells, including self-reactive B cells, in SR-BI^-/-^ mice.

## DISCUSSION

Lipid metabolism has emerged as an important regulator of diverse virus infections, affecting virus replication and the immune response of the host. To date, studies of lipid metabolism in the context of gammaherpesvirus infection have primarily focused on the endogenous lipid synthesis pathways. Importantly, circulating lipid species, including those packaged into lipoproteins, affect both lipid metabolism of the host and cell signaling pathways activated downstream of lipid and lipoprotein receptors. The current study focuses on SR-BI, the primary HDL receptor, and uses a mouse model of SR-BI genetic deficiency. Similar to that observed in humans with SR-BI mutations, lack of global SR-BI expression is associated with an increase in serum HDL levels in mice. Absence of SR-BI expression resulted in increased MHV68 lytic gene expression during the acute stage of MHV68 infection. In contrast, chronic MHV68 infection was associated with exaggerated germinal center response in SR-BI^-/-^ mice and increased differentiation of self-reactive B cells that persisted during the long-term stage of chronic infection. Despite an increased germinal center B cell population observed in MHV68-infected SR-BI^-/-^ mice, these germinal center B cells were less effective at maintaining latent infection.

### SR-BI expression and B cell differentiation

A single published study reported increased B cell activation and proliferation in aged SR-BI^-/-^ mice (69). To our knowledge, the current study is the first to report increased numbers of germinal center B cells of SR-BI^-/-^ mice at baseline and at a young age, with the phenotype further exacerbated by gammaherpesvirus infection. The increase in the germinal center response was also observed upon immunization of SR-BI^-/-^ mice. Thus, infection with the B cell-tropic gammaherpesvirus that drives B cell differentiation had unveiled a previously unknown role of SR-BI as a negative regulator of germinal center responses.

SR-BI is a scavenger receptor: in addition to being a primary HDL receptor, it can also interact with several other host and even microbial ligands, such as LPS and Hepatitis C virus E2 protein (85, 86). Attenuated germinal center response observed in MHV68-infected *Apoa-I^-/-^* mice that express SR-BI but have very low circulating HDL (Fig. 8) suggests that ligation of SR-BI by HDL is not required and may actually interfere with the SR-BI-mediated negative regulation of germinal center response, perhaps by competing with other SR-BI ligands. However, our data do not exclude the possibility that very low levels of HDL observed in *Apoa-I^-/-^* mice were sufficient to attenuate MHV68-driven response or that ligation of another, non-SR-BI receptor by elevated HDL levels in SR-BI^-/-^ mice also contributed to the observed exaggerated germinal center responses.

Given that a mouse model of global SR-BI deficiency was used in this study, the target cell type(s) responsible for the observed germinal center phenotypes remain unknown. In the context of immune populations, SR-BI is highly expressed by macrophages and dendritic cells (immgen.org), potentially affecting the function of antigen-presenting cells that interact with T and B cells to initiate and sustain the germinal center response. However, SR-BI is also expressed by B cells, including germinal center B cells, albeit to a lower level, and could mediate B cell-intrinsic signaling changes that attenuate germinal center responses.

CD4^+^ T follicular helper cells are critical for both physiological and MHV68-driven germinal center responses (23) and were also increased at baseline and following MHV68 infection of SR-BI^-/-^ mice, together with increased MHV68-specific CD8+ T cells in infected SR-BI^-/-^ mice. Importantly, the observed increases in T and B cell activation and differentiation in MHV68-infected SR-BI^-/-^ mice were not driven by the paucity of regulatory CD4+ T cells (Fig. 7). Interestingly, global SR-BI deficiency was previously reported to attenuate thymic T cell development, with decreased numbers of naïve CD4 and CD8 T cells observed at baseline in SR-BI^-/-^ mice (87). In contrast, we did not observe a decrease in total CD8+ and CD4+ T cells in the spleens of naïve SR-BI^-/-^ mice maintained on chow (Fig. 7C, data not shown). Future studies are needed to determine the extent to which T cell-intrinsic or extrinsic SR-BI expression modifies T cell activation and differentiation during immunization or virus infection.

Aged (>30 weeks) SR-BI^-/-^ mice develop self-reactive antibodies and autoimmune disease (69). We found that gammaherpesvirus infection of young SR-BI^-/-^ mice that had undetectable levels of anti-dsDNA antibodies at baseline was sufficient to induce self-reactive antibody generation above that seen in control mice throughout chronic infection. Gammaherpesvirus-driven sustained induction of self-reactive antibodies would be predicted to accelerate the development of autoimmune disease in SR-BI^-/-^ mice, to be tested in future studies. Interestingly, some SR-BI mutations in humans are associated with atherosclerosis (51, 52), despite elevated levels of HDL-C that are typically thought to be protective against cardiovascular disease. Although the mechanisms underlying increased atherosclerosis in humans with SR-BI mutations are still being defined, germinal center responses, including generation of class-switched IgG antibodies, are atherogenic (88, 89). Neither the prevalence of other autoimmune diseases nor B cell responses to common vaccines has been formally examined in humans with loss-of-function SR-BI mutations.

### SR-BI expression and gammaherpesvirus infection

In this study, we observed stage of infection-dependent effects of global SR-BI expression. Specifically, MHV68 lytic gene expression was elevated in the lungs of acutely infected SR-BI^-/-^ mice (Fig. 1). Type II pneumocytes and lung macrophages express high levels of SR-BI (90), and the observed increase in MHV68 lytic gene expression could be a reflection of SR-BI deficiency in these cell types. Importantly, increased MHV68 lytic gene expression in acutely infected SR-BI^-/-^ lungs implies that SR-BI is unlikely to play a role in MHV68 entry, in contrast to that identified for Hepatitis C virus. It is curious that a significant increase in MHV68 lytic gene expression did not translate into a similar increase in the MHV68 lung titers. It is possible that the rising MHV68-specific CD8 T cell response (Fig. 7D and E) had led to the elimination of infected cells in the SR-BI^-/-^ lungs prior to the completion of the entire viral replication cycle. Finally, similar expression of ISGs in acutely infected lungs of control and SR-BI^-/-^ mice suggests that global SR-BI expression does not affect MHV68-driven IFN responses.

Interestingly, we observed decreased frequency of MHV68-infected germinal center B cells in SR-BI^-/-^ spleens. This decreased efficiency of infection occurred despite a significant increase in the overall germinal center B cell population in MHV68-infected SR-BI^-/-^ mice. Importantly, increased levels of germinal center B cells do not always translate to decreased ability of MHV68 to maintain latent infection. We showed that despite a significant increase in germinal center B cells of *Irf1^-/-^* mice, the frequency of MHV68-infected germinal center B cells was similar in control and *Irf1^-/-^* spleens (70). Future studies will be needed to define SR-BI-dependent changes in metabolism and signaling in germinal center B cells that account for the observed antiviral phenotype.

## MATERIALS AND METHODS

### Animals

C57BL/6 J mice (referred to as BL6) were obtained from Jackson Laboratories (stock number 00064, Bar Harbor, ME). *Scarb1^-/-^* mice were obtained from Monty Krieger (MIT), and breeders were fed a chow diet containing 0.5% probucol to enable breeding (91). Weanlings from all mouse strains were maintained on a chow diet starting from 3 weeks of age and until the completion of studies. *ApoA-I^-/-^* mice (59) were obtained from Jackson Laboratories (stock number 2055). All mouse strains were bred and housed in a specific pathogen-free facility at MCW. At 6–7 weeks of age, mice were intranasally infected under light anesthesia with 10,000 plaque-forming units (PFU) of MHV68 or MHV68.ORF73βla (71) diluted in sterile, serum-free Dulbecco’s modified Eagle’s medium (DMEM) immediately prior to infection. MHV68 viral stock was prepared and titered using the 3T12 fibroblast cell line.

### Acute infection studies

Lungs were harvested from individual animals in 1 mL of Dulbecco’s modified Eagle medium (DMEM) containing 10% fetal calf serum (FCS) and mechanically disrupted using 1 mm sterile zirconia beads (catalogue number 9835, RPI Research Products, Mount Prospect, IL) in the Magnalyzer (Roche). Lung homogenates were titered using the 3T12 fibroblast cell line.

### Limiting dilution assays

The frequency of MHV68 DNA-positive cells was determined as previously described (92). Specifically, splenocytes and peritoneal cells were pooled from all mice in each experimental group (3–5 mice/group), and six, 3-fold serial dilutions were subjected to a nested PCR reaction (12 replicates/dilution) using primers against the viral genome. To determine the frequency of *ex vivo* MHV68 reactivation, splenocytes and peritoneal cells were pooled from all mice within each experimental group (3–5 mice/group), and eight, 2-fold serial dilutions of cell suspensions from each group were plated onto a monolayer of MEFs at 24 replicates per dilution. To control for preformed virus four, 2-fold serial dilutions of mechanically disrupted splenic and peritoneal cells were plated on MEFs. Viral reactivation, as indicated by cytopathic clearing of MEFs, was assessed on day 21 of culture.

### qRT-PCR

Individual lungs harvested from acutely infected mice were homogenized in 1 mL of Trizol reagent (Invitrogen) using 1 mm zirconia beads (catalog number 9835, RPI Research Products, Mount Prospect, IL) in the Magnalyzer (Roche). RNA was isolated per manufacturer’s instructions, DNAse-treated, reverse-transcribed, and analyzed by quantitative reverse transcription-PCR (qRT-PCR) using the CFX Connect system (Bio-Rad, Hercules, CA) as previously described (93). Minus RT reactions were used as controls for genomic DNA contamination. Primers were validated for specificity and used within the corresponding linear range. The following primer sequences were used with relative expression quantified by delta-delta Ct method against housekeeping Gapdh expression:

Gapdh Forward (5′-TGTGATGGGTGTGAACCACGAGAA-3′); Gapdh Reverse (5′-GAGCCCTTCCACAATGCCAAAGTT-3′); ORF50 Forward (5′-AGAAACCCACAGCTCGCACTT-3′); ORF50 Reverse (5′-CAATATGCTGGACAGGCGTATC-3′); ORF6 Forward (5′-GTTGCCAGATATCCCTAGGATGA-3′); ORF6 Reverse (5′-ACCTGGCTGGGTCAAGAGACT-3′); Mx1 Forward (5′-AGCTAGACAGAGCAAACCAAGCCA-3′); Mx1 Reverse (5′-TCCCTGAAGCAGACACAGCTGAAA-3′); Ifih Forward (5′-GGAGTGGACAATGGCACAACATCA-3′); Ifih Reverse (5′-TGACGAAACTGTGGTCTCCACACA-3′); Cxcl9 Forward (5’- GCTCTAGAGAGGAGGTCTGATG-3′); and Cxcl9 Reverse (5’- AGGCTTTGGCTAGTCGTTATG-3′)

### Flow cytometry

Single-cell suspensions of total splenocytes or peritoneal exudate cells were prepared in fluorescence-activated cell sorting (FACs) buffer (phosphate-buffered saline, 2% fetal bovine serum); 2 × 10^6^ cells were pre-stained with Fc block and then incubated with optimized antibody concentrations. Data acquisition was performed using a Celesta conventional flow cytometer (BD Biosciences, Franklin Lakes, NJ), and data were analyzed with FlowJo software (BD Life Sciences, Franklin Lakes, NJ). The following list of antibodies used in this study were purchased from BioLegend (San Diego, CA): B220-PECy7 (cat. 103222), CD19-PB (cat. 115523), GL7-APC (cat. 144617), GL7-FITC (cat. 144604), CD95-APC (cat. 152604), CD95-PE (cat. 152608), CD3-PB (cat. 100214), CD4-FITC (cat. 100406), CD8-FITC (cat. 100706), PD-1-BV605 (cat. 135220), CXCR5-PE/Dazzle 594 (cat. 145522), CD44-PECy7 (cat. 103030), Alexa Fluor 647-FoxP3 (cat: 126408); or BD Biosciences (Franklin Lakes, NJ): CD95-PE-CF594 (cat. 562395); or NIH: orf6-APC (cat. 13171). NIH Tetramer core provided the MHC-I tetramers. Cells infected with the MHV68.ORF73bla reporter virus were detected using the LiveBLAzer FRET-B/G Loading Kit with CCF2-AM (cat. K1032) from Thermo Fisher Scientific (Waltham, MA).

### Enzyme-linked immunosorbent assays

ELISAs to measure serum concentrations of total IgM, total IgG, MHV68-specific IgG, and anti-double-stranded DNA IgG were performed as previously described (94)

### Serum cholesterol assay

Serum cholesterol was measured using the Total Cholesterol Kit (catalog number 999-02601, Fujifilm, Lexington, MA) according to the manufacturer’s instructions.

### Immunization

Naive BL6 and SRBI^-/-^ mice were injected intraperitoneally with 300 µL of defibrinated sheep blood (Colorado Serum Company). Spleens were harvested between days 7 and 9 postimmunization, and germinal center responses were assessed by flow cytometry.

### Statistical analyses

Statistical analyses were performed using Student’s *t* test (Prism, GraphPad Software, Inc.).

## Data Availability

All data associated with this study are presented in the figures.

## SUPPLEMENTAL MATERIAL

Supplemental material jvi.00757-25-s0001.pdf Figures S1 to S3.

ASM does not own the copyrights to Supplemental Material that may be linked to, or accessed through, an article. The authors have granted ASM a non-exclusive, world-wide license to publish the Supplemental Material files. Please contact the corresponding author directly for reuse.

## References

[1] Cesarman E. 2014. Gammaherpesviruses and lymphoproliferative disorders. Annu Rev Pathol 9:349–372. doi:10.1146/annurev-pathol-012513-104656 24111911

[2] Bjornevik K, Cortese M, Healy BC, Kuhle J, Mina MJ, Leng Y, Elledge SJ, Niebuhr DW, Scher AI, Munger KL, Ascherio A. 2022. Longitudinal analysis reveals high prevalence of Epstein-Barr virus associated with multiple sclerosis. Science 375:296–301. doi:10.1126/science.abj8222 35025605

[3] Campbell TB, Borok M, Gwanzura L, MaWhinney S, White IE, Ndemera B, Gudza I, Fitzpatrick L, Schooley RT. 2000. Relationship of human herpesvirus 8 peripheral blood virus load and Kaposi's sarcoma clinical stage. AIDS 14:2109–2116. doi:10.1097/00002030-200009290-00006 11061651

[4] Meerbach A, Wutzler P, Häfer R, Zintl F, Gruhn B. 2008. Monitoring of Epstein-Barr virus load after hematopoietic stem cell transplantation for early intervention in post-transplant lymphoproliferative disease. J Med Virol 80:441–454. doi:10.1002/jmv.21096 18205222

[5] Feng W, Cohen JI, Fischer S, Li L, Sneller M, Goldbach-Mansky R, Raab-Traub N, Delecluse H-J, Kenney SC. 2004. Reactivation of latent Epstein-Barr virus by methotrexate: a potential contributor to methotrexate-associated lymphomas. J Natl Cancer Inst 96:1691–1702. doi:10.1093/jnci/djh313 15547182

[6] Orlandi E, Paulli M, Viglio A, Pagnucco G, Riboni R, Baldanti F, Lazzarino M. 2001. Epstein-Barr virus-positive aggressive lymphoma as a consequence of immunosuppression after multiple salvage treatments for follicular lymphoma. Br J Haematol 112:373–376. doi:10.1046/j.1365-2141.2001.02579.x 11167832

[7] Ma SD, Hegde S, Young KH, Sullivan R, Rajesh D, Zhou Y, Jankowska-Gan E, Burlingham WJ, Sun X, Gulley ML, Tang W, Gumperz JE, Kenney SC. 2011. A new model of Epstein-Barr virus infection reveals an important role for early lytic viral protein expression in the development of lymphomas. J Virol 85:165–177. doi:10.1128/JVI.01512-10 20980506 PMC3014199

[8] Bristol JA, Djavadian R, Albright ER, Coleman CB, Ohashi M, Hayes M, Romero-Masters JC, Barlow EA, Farrell PJ, Rochford R, Kalejta RF, Johannsen EC, Kenney SC. 2018. A cancer-associated Epstein-Barr virus BZLF1 promoter variant enhances lytic infection. PLoS Pathog 14:e1007179. doi:10.1371/journal.ppat.1007179 30052684 PMC6082571

[9] Thorley-Lawson DA, Gross A. 2004. Persistence of the Epstein-Barr virus and the origins of associated lymphomas. N Engl J Med 350:1328–1337. doi:10.1056/NEJMra032015 15044644

[10] Efstathiou S, Ho YM, Minson AC. 1990. Cloning and molecular characterization of the murine herpesvirus 68 genome. J Gen Virol 71:1355–1364. doi:10.1099/0022-1317-71-6-1355 2351958

[11] Efstathiou S, Ho YM, Hall S, Styles CJ, Scott SD, Gompels UA. 1990. Murine herpesvirus 68 is genetically related to the gammaherpesviruses Epstein-Barr virus and herpesvirus saimiri. J Gen Virol 71:1365–1372. doi:10.1099/0022-1317-71-6-1365 2161903

[12] Virgin HW, Latreille P, Wamsley P, Hallsworth K, Weck KE, Dal Canto AJ, Speck SH. 1997. Complete sequence and genomic analysis of murine gammaherpesvirus 68. J Virol 71:5894–5904. doi:10.1128/jvi.71.8.5894-5904.1997 9223479 PMC191845

[13] Weck KE, Kim SS, Virgin HW, Speck SH. 1999. Macrophages are the major reservoir of latent murine gammaherpesvirus 68 in peritoneal cells. J Virol 73:3273–3283. doi:10.1128/JVI.73.4.3273-3283.1999 10074181 PMC104091

[14] Li H, Ikuta K, Sixbey JW, Tibbetts SA. 2008. A replication-defective gammaherpesvirus efficiently establishes long-term latency in macrophages but not in B cells in vivo. J Virol 82:8500–8508. doi:10.1128/JVI.00186-08 18562537 PMC2519690

[15] Rekow MM, Darrah EJ, Mboko WP, Lange PT, Tarakanova VL. 2016. Gammaherpesvirus targets peritoneal B-1 B cells for long-term latency. Virology (Auckl) 492:140–144. doi:10.1016/j.virol.2016.02.022 PMC4826794 26945150

[16] Shimakage M, Kimura M, Yanoma S, Ibe M, Yokota S, Tsujino G, Kozuka T, Dezawa T, Tamura S, Ohshima A, Yutsudo M, Hakura A. 1999. Expression of latent and replicative-infection genes of Epstein-Barr virus in macrophage. Arch Virol 144:157–166. doi:10.1007/s007050050492 10076516

[17] Gregory SM, Wang L, West JA, Dittmer DP, Damania B. 2012. Latent Kaposi’s sarcoma-associated herpesvirus infection of monocytes downregulates expression of adaptive immune response costimulatory receptors and proinflammatory cytokines. J Virol 86:3916–3923. doi:10.1128/JVI.06437-11 22278234 PMC3302530

[18] Rappocciolo G, Jenkins FJ, Hensler HR, Piazza P, Jais M, Borowski L, Watkins SC, Rinaldo CR Jr. 2006. DC-SIGN is a receptor for human herpesvirus 8 on dendritic cells and macrophages. J Immunol 176:1741–1749. doi:10.4049/jimmunol.176.3.1741 16424204

[19] Wang LX, Kang G, Kumar P, Lu W, Li Y, Zhou Y, Li Q, Wood C. 2014. Humanized-BLT mouse model of Kaposi's sarcoma-associated herpesvirus infection. Proc Natl Acad Sci USA 111:3146–3151. doi:10.1073/pnas.1318175111 24516154 PMC3939909

[20] Collins CM, Boss JM, Speck SH. 2009. Identification of infected B-cell populations by using a recombinant murine gammaherpesvirus 68 expressing a fluorescent protein. J Virol 83:6484–6493. doi:10.1128/JVI.00297-09 19386718 PMC2698576

[21] Roughan JE, Thorley-Lawson DA. 2009. The intersection of Epstein-Barr virus with the germinal center. J Virol 83:3968–3976. doi:10.1128/JVI.02609-08 19193789 PMC2663245

[22] Tarakanova VL, Suarez F, Tibbetts SA, Jacoby MA, Weck KE, Hess JL, Speck SH, Virgin HW 4th. 2005. Murine gammaherpesvirus 68 infection is associated with lymphoproliferative disease and lymphoma in BALB β2 microglobulin-deficient mice. J Virol 79:14668–14679. doi:10.1128/JVI.79.23.14668-14679.2005 16282467 PMC1287585

[23] Collins CM, Speck SH. 2014. Expansion of murine gammaherpesvirus latently infected B cells requires T follicular help. PLoS Pathog 10:e1004106. doi:10.1371/journal.ppat.1004106 24789087 PMC4006913

[24] Collins CM, Speck SH. 2012. Tracking murine gammaherpesvirus 68 infection of germinal center B cells in vivo. PLoS One 7:e33230. doi:10.1371/journal.pone.0033230 22427999 PMC3302828

[25] Thorley-Lawson DA. 2001. Epstein-Barr virus: exploiting the immune system. Nat Rev Immunol 1:75–82. doi:10.1038/35095584 11905817

[26] Johnson KE, Tarakanova VL. 2020. Gammaherpesviruses and B cells: a relationship that lasts a lifetime. Viral Immunol 33:316–326. doi:10.1089/vim.2019.0126 31913773 PMC7247026

[27] Flaño E, Kim I-J, Woodland DL, Blackman MA. 2002. Gamma-herpesvirus latency is preferentially maintained in splenic germinal center and memory B cells. J Exp Med 196:1363–1372. doi:10.1084/jem.20020890 12438427 PMC2193987

[28] Laichalk LL, Thorley-Lawson DA. 2005. Terminal differentiation into plasma cells initiates the replicative cycle of Epstein-Barr virus in vivo. J Virol 79:1296–1307. doi:10.1128/JVI.79.2.1296-1307.2005 15613356 PMC538585

[29] Liang X, Collins CM, Mendel JB, Iwakoshi NN, Speck SH. 2009. Gammaherpesvirus-driven plasma cell differentiation regulates virus reactivation from latently infected B lymphocytes. PLoS Pathog 5:e1000677. doi:10.1371/journal.ppat.1000677 19956661 PMC2777334

[30] Jondle CN, Johnson KE, Aurubin C, Sylvester P, Xin G, Cui W, Huppler AR, Tarakanova VL. 2021. Gammaherpesvirus usurps host IL-17 signaling to support the establishment of chronic infection. mBio 12:e00566-21. doi:10.1128/mBio.00566-21 33824206 PMC8092251

[31] Lee J, Cullum E, Stoltz K, Bachmann N, Strong Z, Millick DD, Denzin LK, Chang A, Tarakanova V, Chervonsky AV, Golovkina T. 2021. Mouse homologue of human HLA-DO does not preempt autoimmunity but controls murine gammaherpesvirus MHV68. J Immunol 207:2944–2951. doi:10.4049/jimmunol.2100650 34810225 PMC9124240

[32] Darrah EJ, Jondle CN, Johnson KE, Xin G, Lange PT, Cui W, Olteanu H, Tarakanova VL. 2019. Conserved gammaherpesvirus protein kinase selectively promotes irrelevant B cell responses. J Virol 93:e01760-18. doi:10.1128/JVI.01760-18 30728267 PMC6450124

[33] Johnson KE, Lange PT, Jondle CN, Volberding PJ, Lorenz UM, Cui W, Dittel BN, Tarakanova VL. 2019. B cell-intrinsic SHP1 expression promotes the gammaherpesvirus-driven germinal center response and the establishment of chronic infection. J Virol 94:e01232-19. doi:10.1128/JVI.01232-19 31597758 PMC6912115

[34] Mboko WP, Olteanu H, Ray A, Xin G, Darrah EJ, Kumar SN, Kulinski JM, Cui W, Dittel BN, Gauld SB, Tarakanova VL. 2015. Tumor suppressor interferon-regulatory factor 1 counteracts the germinal center reaction driven by a cancer-associated gammaherpesvirus. J Virol 90:2818–2829. doi:10.1128/JVI.02774-15 26719266 PMC4810652

[35] Terrell S, Speck SH. 2017. Murine gammaherpesvirus M2 antigen modulates splenic B cell activation and terminal differentiation in vivo. PLoS Pathog 13:e1006543. doi:10.1371/journal.ppat.1006543 28767707 PMC5555712

[36] Wang Y, Feswick A, Apostolou V, Petkov PM, Moser EK, Tibbetts SA. 2022. Gammaherpesvirus-mediated repression reveals EWSR1 to be a negative regulator of B cell responses. Proc Natl Acad Sci USA 119:e2123362119. doi:10.1073/pnas.2123362119 35921433 PMC9371696

[37] Wang Y, Feldman ER, Bullard WL, Tibbetts SA. 2019. A gammaherpesvirus MicroRNA Targets EWSR1 (Ewing sarcoma breakpoint region 1) in vivo to promote latent infection of germinal center B cells. mBio 10:e00996-19. doi:10.1128/mBio.00996-19 31363027 PMC6667617

[38] Wang Y, Manzi M, Feswick A, Renshaw L, Oliver PM, Tibbetts SA, Moser EK. 2023. B cell expression of E3 ubiquitin ligase Cul4b promotes chronic gammaherpesvirus infection in vivo. J Virol 97:e0100823. doi:10.1128/jvi.01008-23 37962378 PMC10734415

[39] Cieniewicz B, Kirillov V, Daher I, Li X, Oldenburg DG, Dong Q, Bettke JA, Marcu KB, Krug LT. 2022. IKKα-mediated noncanonical NF-κB signaling is required to support murine gammaherpesvirus 68 latency in vivo. J Virol 96:e0002722. doi:10.1128/jvi.00027-22 35481781 PMC9131860

[40] Rodrigues L, Popov N, Kaye KM, Simas JP. 2013. Stabilization of Myc through heterotypic poly-ubiquitination by mLANA is critical for γ-herpesvirus lymphoproliferation. PLoS Pathog 9:e1003554. doi:10.1371/journal.ppat.1003554 23950719 PMC3738482

[41] Kim IJ, Burkum CE, Cookenham T, Schwartzberg PL, Woodland DL, Blackman MA. 2007. Perturbation of B cell activation in SLAM-associated protein-deficient mice is associated with changes in gammaherpesvirus latency reservoirs. J Immunol 178:1692–1701. doi:10.4049/jimmunol.178.3.1692 17237419

[42] Johansen ER, Schmalzriedt DL, Avila D, Sylvester PA, Rahlf CR, Bobek JM, Sahoo D, Dittel BN, Tarakanova VL. 2024. Combination of proviral and antiviral roles of B cell-intrinsic STAT1 expression defines parameters of chronic gammaherpesvirus infection. mBio 15:e0159824. doi:10.1128/mbio.01598-24 39440973 PMC11559066

[43] Sanchez EL, Pulliam TH, Dimaio TA, Thalhofer AB, Delgado T, Lagunoff M. 2017. Glycolysis, glutaminolysis, and fatty acid synthesis are required for distinct stages of Kaposi's sarcoma-associated herpesvirus lytic replication. J Virol 91:e02237-16. doi:10.1128/JVI.02237-16 28275189 PMC5411582

[44] Delgado T, Sanchez EL, Camarda R, Lagunoff M. 2012. Global metabolic profiling of infection by an oncogenic virus: KSHV induces and requires lipogenesis for survival of latent infection. PLoS Pathog 8:e1002866. doi:10.1371/journal.ppat.1002866 22916018 PMC3420960

[45] Wang LW, Wang Z, Ersing I, Nobre L, Guo R, Jiang S, Trudeau S, Zhao B, Weekes MP, Gewurz BE. 2019. Epstein-Barr virus subverts mevalonate and fatty acid pathways to promote infected B-cell proliferation and survival. PLoS Pathog 15:e1008030. doi:10.1371/journal.ppat.1008030 31518366 PMC6760809

[46] Lange PT, Schorl C, Sahoo D, Tarakanova VL. 2018. Liver X receptors suppress activity of cholesterol and fatty acid synthesis pathways to oppose gammaherpesvirus replication. mBio 9:e01115-18. doi:10.1128/mBio.01115-18 30018108 PMC6050960

[47] Lange PT, Darrah EJ, Vonderhaar EP, Mboko WP, Rekow MM, Patel SB, Sidjanin DJ, Tarakanova VL. 2016. Type I interferon counteracts antiviral effects of statins in the context of gammaherpesvirus infection. J Virol 90:3342–3354. doi:10.1128/JVI.02277-15 26739055 PMC4794672

[48] Martin SS, Aday AW, Allen NB, Almarzooq ZI, Anderson CAM, Arora P, Avery CL, Baker-Smith CM, Bansal N, Beaton AZ, et al.. 2025. 2025 heart disease and stroke statistics: a report of US and global data from the American heart association. Circulation 151:e41–e660. doi:10.1161/CIR.0000000000001303 39866113 PMC12256702

[49] Fan J, Watanabe T. 2022. Atherosclerosis: known and unknown. Pathol Int 72:151–160. doi:10.1111/pin.13202 35076127

[50] Thomas SR, Zhang Y, Rye K-A. 2023. The pleiotropic effects of high-density lipoproteins and apolipoprotein A-I. Best Pract Res Clin Endocrinol Metab 37:101689. doi:10.1016/j.beem.2022.101689 36008277

[51] Zanoni P, Khetarpal SA, Larach DB, Hancock-Cerutti WF, Millar JS, Cuchel M, DerOhannessian S, Kontush A, Surendran P, Saleheen D, et al.. 2016. Rare variant in scavenger receptor BI raises HDL cholesterol and increases risk of coronary heart disease. Science 351:1166–1171. doi:10.1126/science.aad3517 26965621 PMC4889017

[52] Koenig SN, Sucharski HC, Jose EM, Dudley EK, Madiai F, Cavus O, Argall AD, Williams JL, Murphy NP, Keith CBR, El Refaey M, Gumina RJ, Boudoulas KD, Milks MW, Sofowora G, Smith SA, Hund TJ, Wright NT, Bradley EA, Zareba KM, Wold LE, Mazzaferri EL Jr, Mohler PJ. 2021. Inherited variants in SCARB1 cause severe early-onset coronary artery disease. Circ Res 129:296–307. doi:10.1161/CIRCRESAHA.120.318793 33975440 PMC8273129

[53] Xu S, Laccotripe M, Huang X, Rigotti A, Zannis VI, Krieger M. 1997. Apolipoproteins of HDL can directly mediate binding to the scavenger receptor SR-BI, an HDL receptor that mediates selective lipid uptake. J Lipid Res 38:1289–1298. doi:10.1016/S0022-2275(20)37413-7 9254056

[54] Powers HR, Sahoo D. 2022. SR-B1's next top model: structural perspectives on the functions of the HDL receptor. Curr Atheroscler Rep 24:277–288. doi:10.1007/s11883-022-01001-1 35107765 PMC8809234

[55] Chadwick AC, Sahoo D. 2013. Functional genomics of the human high-density lipoprotein receptor scavenger receptor BI: an old dog with new tricks. Curr Opin Endocrinol Diabetes Obes 20:124–131. doi:10.1097/MED.0b013e32835ed575 23403740 PMC3967407

[56] Rigotti A, Trigatti BL, Penman M, Rayburn H, Herz J, Krieger M. 1997. A targeted mutation in the murine gene encoding the high density lipoprotein (HDL) receptor scavenger receptor class B type I reveals its key role in HDL metabolism. Proc Natl Acad Sci USA 94:12610–12615. doi:10.1073/pnas.94.23.12610 9356497 PMC25055

[57] Van Eck M, Twisk J, Hoekstra M, Van Rij BT, Van der Lans CAC, Bos IST, Kruijt JK, Kuipers F, Van Berkel TJC. 2003. Differential effects of scavenger receptor BI deficiency on lipid metabolism in cells of the arterial wall and in the liver. J Biol Chem 278:23699–23705. doi:10.1074/jbc.M211233200 12639961

[58] Fuller M, Dadoo O, Serkis V, Abutouk D, MacDonald M, Dhingani N, Macri J, Igdoura SA, Trigatti BL. 2014. The effects of diet on occlusive coronary artery atherosclerosis and myocardial infarction in scavenger receptor class B, type 1/low-density lipoprotein receptor double knockout mice. Arterioscler Thromb Vasc Biol 34:2394–2403. doi:10.1161/ATVBAHA.114.304200 25212235

[59] Williamson R, Lee D, Hagaman J, Maeda N. 1992. Marked reduction of high density lipoprotein cholesterol in mice genetically modified to lack apolipoprotein A-I. Proc Natl Acad Sci USA 89:7134–7138. doi:10.1073/pnas.89.15.7134 1496008 PMC49660

[60] Tibbetts SA, Loh J, van Berkel V, McClellan JS, Jacoby MA, Kapadia SB, Speck SH, Virgin HW IV. 2003. Establishment and maintenance of gammaherpesvirus latency are independent of infective dose and route of infection. J Virol 77:7696–7701. doi:10.1128/JVI.77.13.7696-7701.2003 12805472 PMC164792

[61] Dutia BM, Allen DJ, Dyson H, Nash AA. 1999. Type I interferons and IRF-1 play a critical role in the control of a gammaherpesvirus infection. Virology (Auckl) 261:173–179. doi:10.1006/viro.1999.9834 10497103

[62] Barton ES, Lutzke ML, Rochford R, Virgin HW. 2005. Alpha/beta interferons regulate murine gammaherpesvirus latent gene expression and reactivation from latency. J Virol 79:14149–14160. doi:10.1128/JVI.79.22.14149-14160.2005 16254350 PMC1280204

[63] Goodwin MM, Canny S, Steed A, Virgin HW. 2010. Murine gammaherpesvirus 68 has evolved gamma interferon and stat1-repressible promoters for the lytic switch gene 50. J Virol 84:3711–3717. doi:10.1128/JVI.02099-09 20071569 PMC2838114

[64] Wood BM, Mboko WP, Mounce BC, Tarakanova VL. 2013. Mouse gammaherpesvirus-68 infection acts as a rheostat to set the level of type I interferon signaling in primary macrophages. Virology (Auckl) 443:123–133. doi:10.1016/j.virol.2013.04.036 PMC3703304 23706314

[65] Vasquez M, Fioravanti J, Aranda F, Paredes V, Gomar C, Ardaiz N, Fernandez-Ruiz V, Méndez M, Nistal-Villan E, Larrea E, Gao Q, Gonzalez-Aseguinolaza G, Prieto J, Berraondo P. 2016. Interferon alpha bioactivity critically depends on scavenger receptor class B type I function. Oncoimmunology 5:e1196309. doi:10.1080/2162402X.2016.1196309 27622065 PMC5007953

[66] Liu SY, Sanchez DJ, Aliyari R, Lu S, Cheng G. 2012. Systematic identification of type I and type II interferon-induced antiviral factors. Proc Natl Acad Sci USA 109:4239–4244. doi:10.1073/pnas.1114981109 22371602 PMC3306696

[67] Tan CSE, Lawler C, May JS, Belz GT, Stevenson PG. 2016. Type I interferons direct gammaherpesvirus host colonization. PLoS Pathog 12:e1005654. doi:10.1371/journal.ppat.1005654 27223694 PMC4880296

[68] Collins CM, Speck SH. 2015. Interleukin 21 signaling in B cells is required for efficient establishment of murine gammaherpesvirus latency. PLoS Pathog 11:e1004831. doi:10.1371/journal.ppat.1004831 25875847 PMC4395336

[69] Feng H, Guo L, Wang D, Gao H, Hou G, Zheng Z, Ai J, Foreman O, Daugherty A, Li XA. 2011. Deficiency of scavenger receptor BI leads to impaired lymphocyte homeostasis and autoimmune disorders in mice. Arterioscler Thromb Vasc Biol 31:2543–2551. doi:10.1161/ATVBAHA.111.234716 21836069 PMC3202973

[70] Mboko WP, Olteanu H, Ray A, Xin G, Darrah EJ, Kumar SN, Kulinski JM, Cui W, Dittel BN, Gauld SB, Tarakanova VL. 2016. Tumor suppressor interferon-regulatory factor 1 counteracts the germinal center reaction driven by a cancer-associated gammaherpesvirus. J Virol 90:2818–2829. doi:10.1128/JVI.02774-15 PMC4810652 26719266

[71] Nealy MS, Coleman CB, Li H, Tibbetts SA. 2010. Use of a virus-encoded enzymatic marker reveals that a stable fraction of memory B cells expresses latency-associated nuclear antigen throughout chronic gammaherpesvirus infection. J Virol 84:7523–7534. doi:10.1128/JVI.02572-09 20484501 PMC2897616

[72] Sangster MY, Topham DJ, D’Costa S, Cardin RD, Marion TN, Myers LK, Doherty PC. 2000. Analysis of the virus-specific and nonspecific B cell response to a persistent B-lymphotropic gammaherpesvirus. J Immunol 164:1820–1828. doi:10.4049/jimmunol.164.4.1820 10657630

[73] Gauld SB, De Santis JL, Kulinski JM, McGraw JA, Leonardo SM, Ruder EA, Maier W, Tarakanova VL. 2013. Modulation of B-cell tolerance by murine gammaherpesvirus 68 infection: requirement for Orf73 viral gene expression and follicular helper T cells. Immunology 139:197–204. doi:10.1111/imm.12069 23311955 PMC3647186

[74] Tracy SI, Kakalacheva K, Lünemann JD, Luzuriaga K, Middeldorp J, Thorley-Lawson DA. 2012. Persistence of Epstein-Barr virus in self-reactive memory B cells. J Virol 86:12330–12340. doi:10.1128/JVI.01699-12 22951828 PMC3486485

[75] Decalf J, Godinho-Silva C, Fontinha D, Marques S, Simas JP. 2014. Establishment of murine gammaherpesvirus latency in B cells is not a stochastic event. PLoS Pathog 10:e1004269. doi:10.1371/journal.ppat.1004269 25079788 PMC4117635

[76] Fleisher GR, Collins M, Fager S. 1983. Limitations of available tests for diagnosis of infectious mononucleosis. J Clin Microbiol 17:619–624. doi:10.1128/jcm.17.4.619-624.1983 6304142 PMC272704

[77] Stevenson PG, Doherty PC. 1998. Kinetic analysis of the specific host response to a murine gammaherpesvirus. J Virol 72:943–949. doi:10.1128/JVI.72.2.943-949.1998 9444986 PMC124564

[78] Temel RE, Trigatti B, DeMattos RB, Azhar S, Krieger M, Williams DL. 1997. Scavenger receptor class B, type I (SR-BI) is the major route for the delivery of high density lipoprotein cholesterol to the steroidogenic pathway in cultured mouse adrenocortical cells. Proc Natl Acad Sci USA 94:13600–13605. doi:10.1073/pnas.94.25.13600 9391072 PMC28352

[79] Lai WA, Yeh YT, Lee MT, Wu LS, Ke FC, Hwang JJ. 2013. Ovarian granulosa cells utilize scavenger receptor SR-BI to evade cellular cholesterol homeostatic control for steroid synthesis. J Lipid Res 54:365–378. doi:10.1194/jlr.M030239 23197320 PMC3588866

[80] Gowdy KM, Madenspacher JH, Azzam KM, Gabor KA, Janardhan KS, Aloor JJ, Fessler MB. 2015. Key role for scavenger receptor B-I in the integrative physiology of host defense during bacterial pneumonia. Mucosal Immunol 8:559–571. doi:10.1038/mi.2014.88 25336169 PMC4406784

[81] Kim D, Nguyen QT, Lee J, Lee SH, Janocha A, Kim S, Le HT, Dvorina N, Weiss K, Cameron MJ, Asosingh K, Erzurum SC, Baldwin WM 3rd, Lee J-S, Min B. 2020. Anti-inflammatory roles of glucocorticoids are mediated by Foxp3^+^ regulatory T cells via a miR-342-dependent mechanism. Immunity 53:581–596. doi:10.1016/j.immuni.2020.07.002 32707034 PMC7793548

[82] Tibbetts SA, van Dyk LF, Speck SH, Virgin HW 4th. 2002. Immune control of the number and reactivation phenotype of cells latently infected with a gammaherpesvirus. J Virol 76:7125–7132. doi:10.1128/jvi.76.14.7125-7132.2002 12072512 PMC136321

[83] Peacock JW, Bost KL. 2001. Murine gammaherpesvirus-68-induced interleukin-10 increases viral burden, but limits virus-induced splenomegaly and leukocytosis. Immunology 104:109–117. doi:10.1046/j.1365-2567.2001.01286.x 11576228 PMC1783283

[84] Camus MC, Chapman MJ, Forgez P, Laplaud PM. 1983. Distribution and characterization of the serum lipoproteins and apoproteins in the mouse, Mus musculus. J Lipid Res 24:1210–1228. doi:10.1016/S0022-2275(20)37904-9 6631247

[85] Vishnyakova TG, Bocharov AV, Baranova IN, Chen Z, Remaley AT, Csako G, Eggerman TL, Patterson AP. 2003. Binding and internalization of lipopolysaccharide by Cla-1, a human orthologue of rodent scavenger receptor B1. J Biol Chem 278:22771–22780. doi:10.1074/jbc.M211032200 12651854

[86] Scarselli E, Ansuini H, Cerino R, Roccasecca RM, Acali S, Filocamo G, Traboni C, Nicosia A, Cortese R, Vitelli A. 2002. The human scavenger receptor class B type I is a novel candidate receptor for the hepatitis C virus. EMBO J 21:5017–5025. doi:10.1093/emboj/cdf529 12356718 PMC129051

[87] Zheng Z, Ai J, Guo L, Ye X, Bondada S, Howatt D, Daugherty A, Li XA. 2018. SR-BI (scavenger receptor class b type 1) is critical in maintaining normal T-cell development and enhancing thymic regeneration. Arterioscler Thromb Vasc Biol 38:2706–2717. doi:10.1161/ATVBAHA.118.311728 30354229 PMC6209104

[88] Centa M, Jin H, Hofste L, Hellberg S, Busch A, Baumgartner R, Verzaal NJ, Lind Enoksson S, Perisic Matic L, Boddul SV, Atzler D, Li DY, Sun C, Hansson GK, Ketelhuth DFJ, Hedin U, Wermeling F, Lutgens E, Binder CJ, Maegdesfessel L, Malin SG. 2019. Germinal center-derived antibodies promote atherosclerosis plaque size and stability. Circulation 139:2466–2482. doi:10.1161/CIRCULATIONAHA.118.038534 30894016

[89] Ebrahimian T, Dierick F, Ta V, Kotsiopriftis M, O’Connor Miranda J, Mann KK, Orthwein A, Lehoux S. 2023. B cell-specific knockout of AID protects against atherosclerosis. Sci Rep 13:8723. doi:10.1038/s41598-023-35980-1 37253865 PMC10229602

[90] Kolleck I, Witt W, Wissel H, Sinha P, Rüstow B. 2000. HDL and vitamin E in plasma and the expression of SR-BI on lung cells during rat perinatal development. Lung 178:191–200. doi:10.1007/s004080000023 10960554

[91] Miettinen HE, Rayburn H, Krieger M. 2001. Abnormal lipoprotein metabolism and reversible female infertility in HDL receptor (SR-BI)-deficient mice. J Clin Invest 108:1717–1722. doi:10.1172/JCI13288 11733567 PMC200987

[92] Weck KE, Barkon ML, Yoo LI, Speck SH, Virgin HW IV. 1996. Mature B cells are required for acute splenic infection, but not for establishment of latency, by murine gammaherpesvirus 68. J Virol 70:6775–6780. doi:10.1128/JVI.70.10.6775-6780.1996 8794315 PMC190721

[93] Sylvester PA, Jondle CN, Stoltz KP, Lanham J, Dittel BN, Tarakanova VL. 2021. Conserved gammaherpesvirus protein kinase counters the antiviral effects of myeloid cell-specific STAT1 expression to promote the establishment of splenic B cell latency. J Virol 95:e0085921. doi:10.1128/JVI.00859-21 34132573 PMC8354328

[94] Jondle CN, Sylvester PA, Schmalzriedt DL, Njoya K, Tarakanova VL. 2022. The antagonism between the murine gammaherpesvirus protein kinase and global interferon regulatory factor 1 expression shapes the establishment of chronic infection. J Virol 96:e0126022. doi:10.1128/jvi.01260-22 36169331 PMC9599343

