# Supplementary material for: Gammaherpesvirus infection unveils exaggerated germinal center responses in an SR-BI-deficient host
Source: J Virol. 2025 May 30;99(7):e00757-25. doi: 10.1128/jvi.00757-25 (PMC12282102; doi:10.1128/jvi.00757-25)

Supplemental Figure Legends:

Supplemental Figure 1. Representative gating strategy for immunophenotyping baseline populations of splenic B cells. FO – follicular B cells, MZ- marginal zone B cells.

Supplemental Figure 2. Splenic B cell immunophenotyping. Splenic B cell populations from naive mice of indicated genotypes and ages were measured using gating strategy in Supplemental Figure 1. Proportions and absolute numbers are shown for each population. Each symbol represents an individual spleen. *$p<0.05$, **$p<0.01$, ***$p<0.001$.

Supplemental Figure 3. Representative gating strategy to detect loading of uncleaved CCF2 in germinal center B cells of mice infected with the MHV68.orf73βla reporter virus. Germinal center B cells were identified using indicated markers in spleens collected at 16 days post infection.

Splenic B cell immunophenotyping, pregated on CD19+

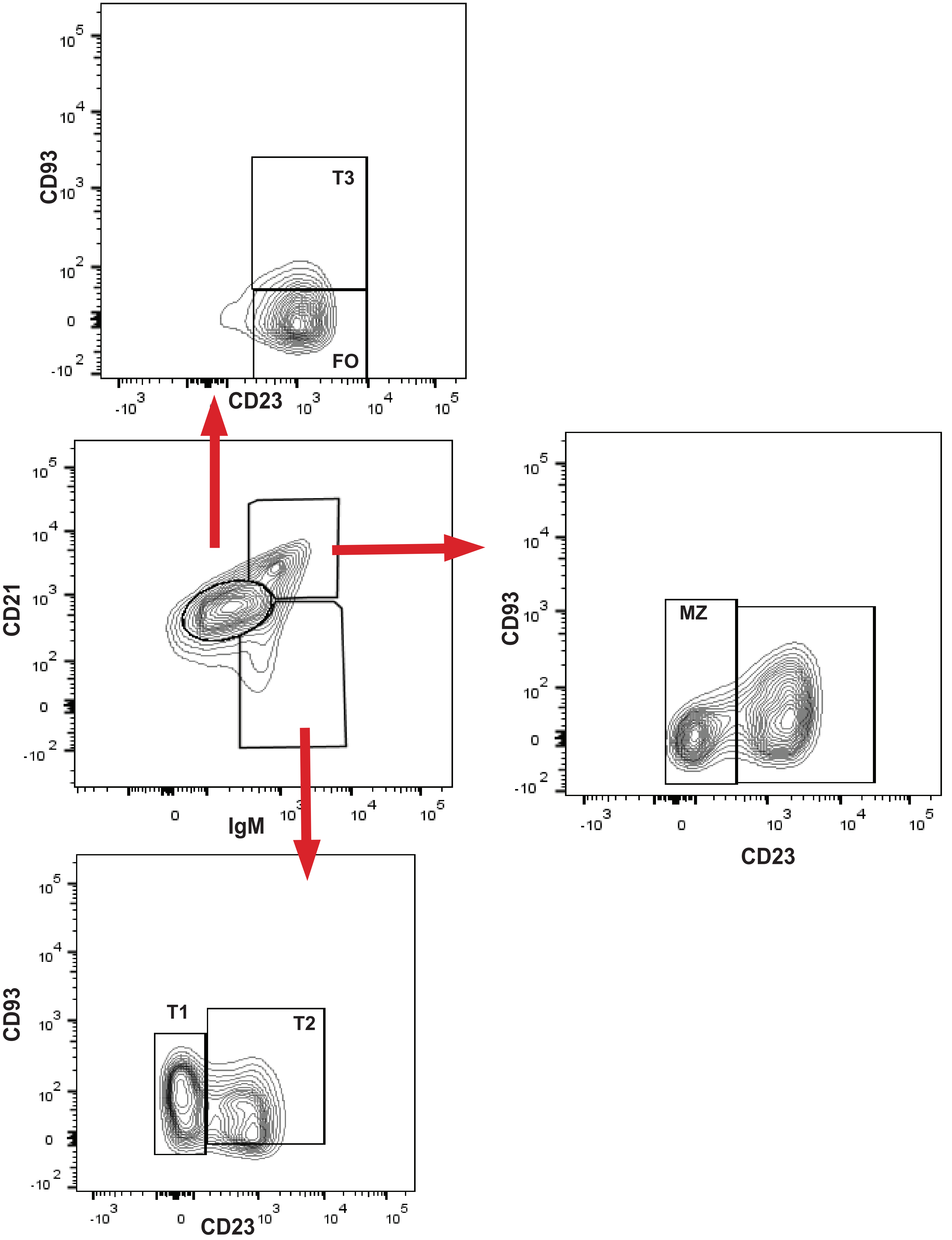

Supplemental Figure 2

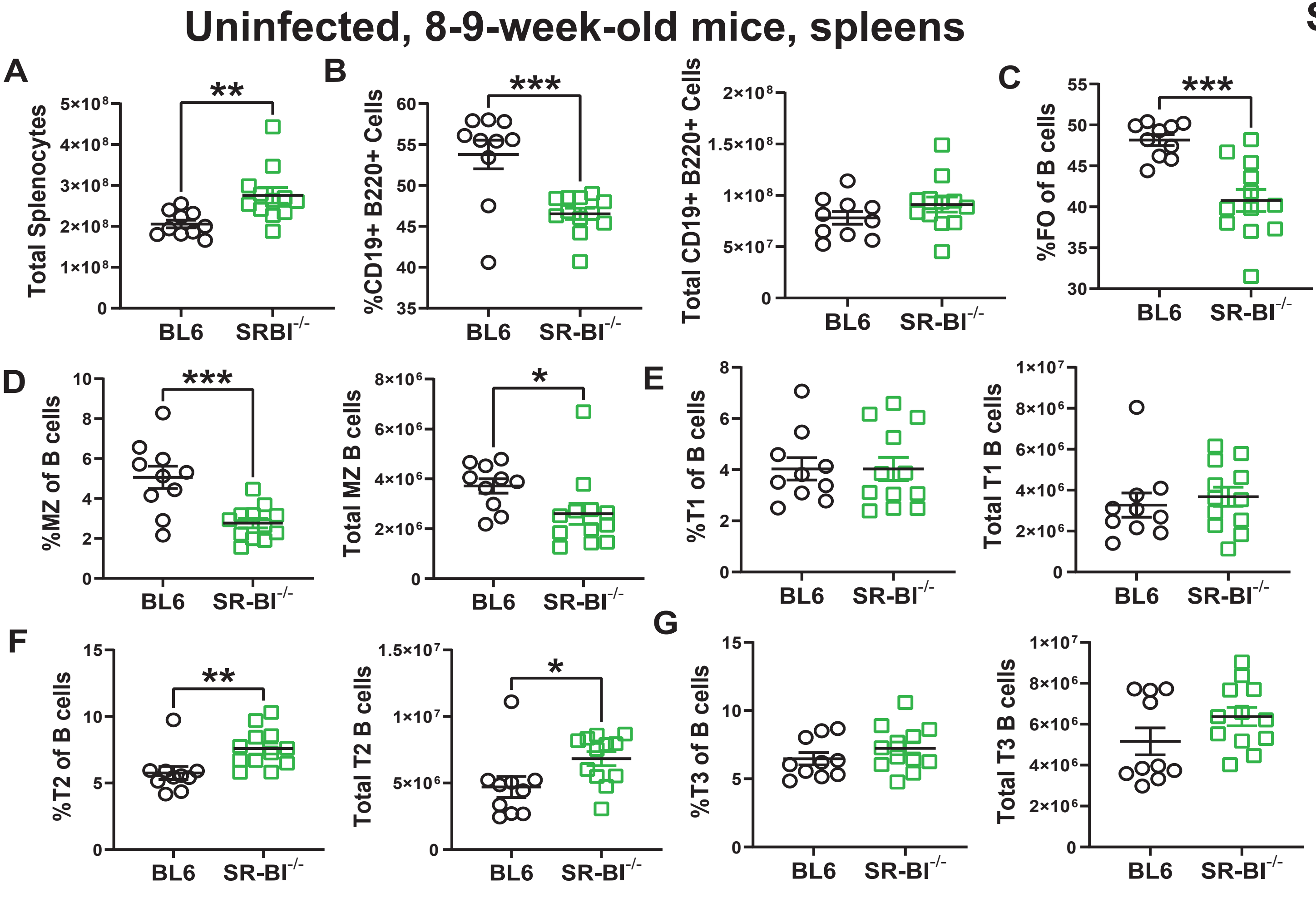

Supplemental Figure 3

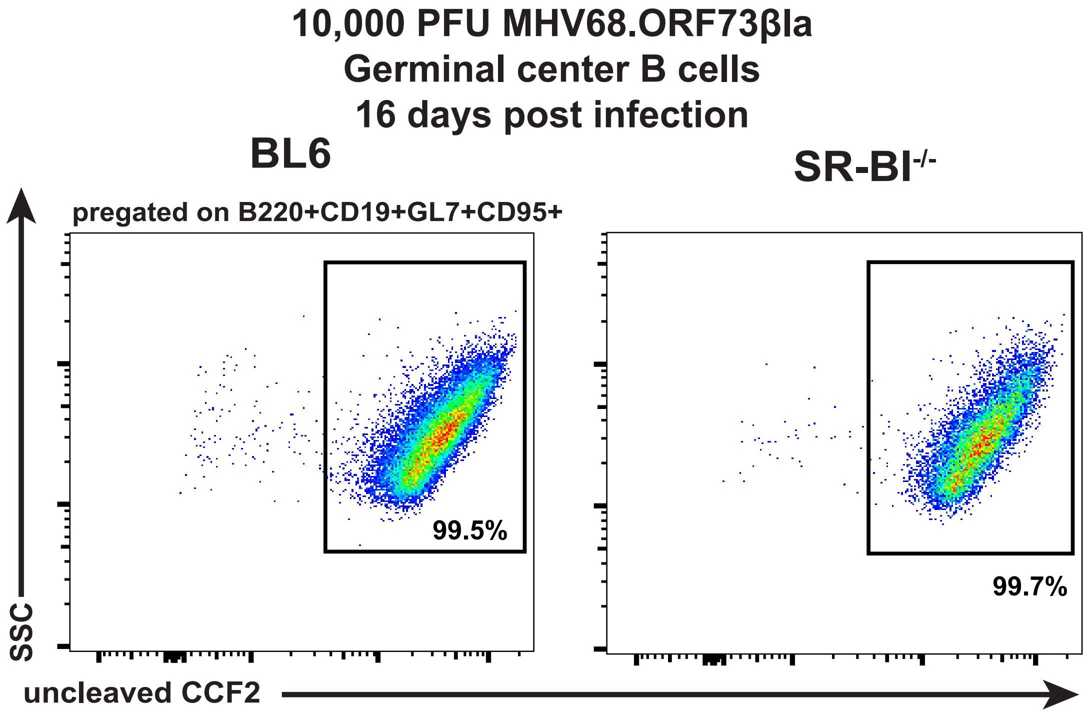

Supplement: Supplemental material — Figures S1 to S3. [file jvi.00757-25-s0001.pdf]
